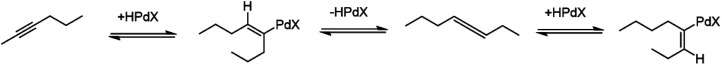# Palladium-catalyzed hydroboration reaction of unactivated alkynes with bis (pinacolato) diboron in water†

**DOI:** 10.1039/d1ra09136k

**Published:** 2022-03-29

**Authors:** Ming Yang, Yunzi Yu, Wenxia Ma, Yuqin Feng, Gang Zhang, Yaqi Wu, Fanyu Zhou, Yongsheng Yang, Dezheng Liu

**Affiliations:** School of Chemistry and Engineering, Hubei Key Laboratory of Biomass Fibers and Eco-dyeing & Finishing, Wuhan Textile University 1 Textile Road Wuhan 430073 Hubei China ysyang@wtu.edu.cn; School of Mechanical Engineering, Hubei University of Arts and Science No. 296 Longzhong Road Xiangyang Hubei Province 41053 P. R. China Liudezheng@126.com

## Abstract

A highly efficient and mild palladium-catalyzed hydroboration of unactivated internal alkynes in water is described. Both aryl- and alkyl-substituted alkynes proceeded smoothly within the reaction time to afford the desired vinylboronates in moderate to high yields. Bis (pinacolato) diboron was used to afford α- and β-hydroborated products in the presence of HOAc. These reactions showed high reactivities and tolerance, thus providing a promising method for the synthesis of alkenyl boron compounds.

## Abstract 2

A highly efficient and mild palladium-catalyzed hydroboration of unactivated internal alkynes in water.

## Graphical abstract

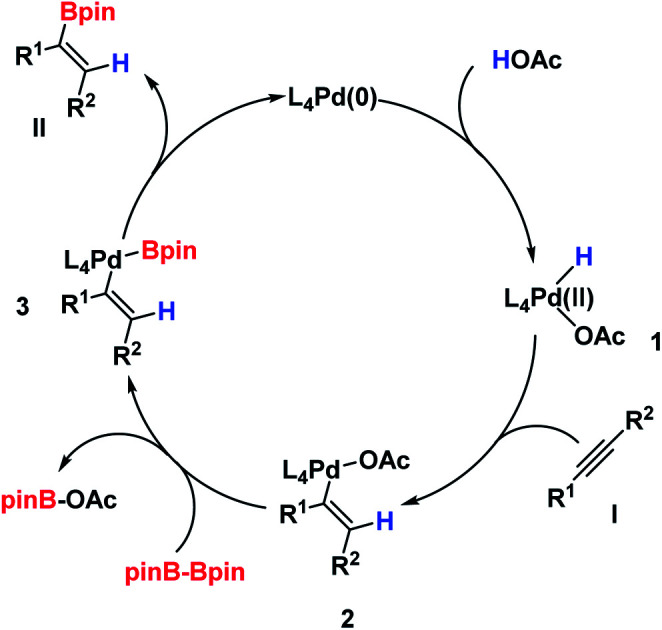

## Introduction

In the past few decades, organoboron compounds have received extensive attention due to their diverse biological activities and important synthetic intermediates in the fields of organic synthesis, materials science and drug development, especially as important synthetic intermediates in organic synthesis, the versatility shown by the structure is very eye-catching.^1,2^ Organoboron compounds could be used as essential carbon nucleophiles for the introduction of functional groups and the C–C bond formation process such as Suzuki–Miyaura coupling and Petasis reaction. The current research provides numerous feasible and effective new methods for preparing organoboron compounds, such as asymmetric catalytic reaction to prepare a chiral borane. In this circumstance, vinyl boronates, which are versatile organic synthetic intermediates, are now gaining increasing attention and have been widely used in various carbon–carbon bond forming reactions.^3^ Compared with the previous methods that used organic halides (I, Br, Cl) as reagents, our method of using organic boron reagents is more effective for the formation of C–C bonds and direct hydroboration reactions. High-yield products can be obtained by the method. The method we developed is very compatible with various functional groups.^4^ The organoboron reagent can be used as a necessary carbon nucleophile in the reaction of introducing functional groups and the formation of C–C bonds (such as Suzuki–Miyaura coupling and Petasis reaction); it is an excellent borohydride reagent.^5^ It is very important to develop synthetic methods for organoboron compounds because these compounds can be directly used in various transformations, such as the construction of C–C bonds and C–B bonds.^6^

**Scheme 1:**
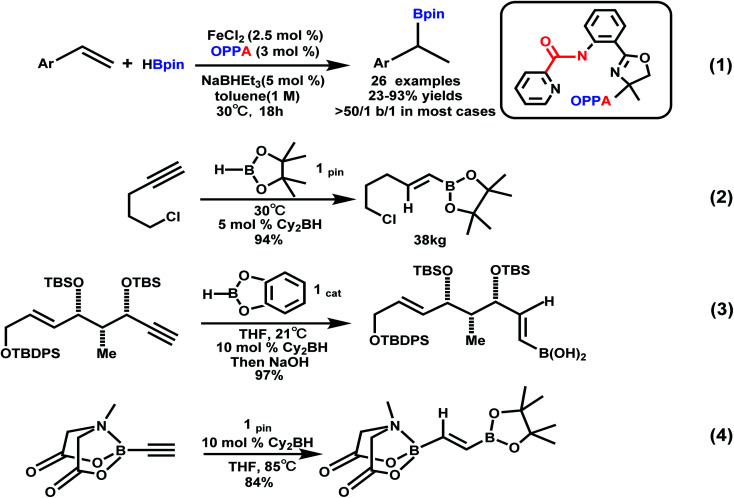
Hydroboration of styrenes and selected applications of Arase–Hoshi hydroboration.^15,17*b*–*d*^

Considerable attention was focused on alkenylboron compounds, whose versatility has been demonstrated by the stereodefined construction of valuable multi-substituted alkenes including biologically active molecules, natural products, and functional materials.^7^ Cobalt,^8^ ruthenium,^9^ aluminum,^10^ copper,^11^ iron,^12^ manganese,^13^ and ytterbium^14^ were used in the hydroboration reaction of alkenes recently. For example, oxazolinyl phenyl picolinic acid amide as a ligand, styrene was subjected to a highly Markovnikov selective hydroboration reaction under iron catalysis to provide the branched borohydride product (Scheme 1, eqn (1)),^15*a*^ and Jiang *et al.* have successfully developed the regioselective palladium(ii)-catalyzed hydroboration of aryl alkenes with B_2_pin_2_.^15*b*^ Alkenyl boron reagents are ubiquitous in synthesis and very important in stereospecific cross-coupling reactions to generate olefins. The hydroboration of alkynes is a useful and concise method for the preparation of alkenyl boranes, which are versatile organic building blocks in subsequent couplings.^16^ For example, Arase and Hoshi reported that at room temperature, in THF, Cy_2_BH and 9-BBN catalyzed the regioselective *cis* hydroboration reaction of alkynes, thereby preparing the *E*-alkenyl pinacol borate in high yields without being polluted by metal catalysts.^17*a*^ In the past two decades, the Arase–Hoshi R_2_BH-catalyzed alkyne hydroboration reaction has been widely used (Scheme 1, eqn (2)–(4)).^17*b*–*d*^ Recently, the groups of Yun Sawamura and Hoveyda have made great contributions to the borohydride reaction with Cu as a catalyst. In the case of adding MeOH, the organoboron reagent combined with α,β-unsaturated carbonyl compounds,^18^ olefins^19^ and alkyne^20^ underwent an addition reaction, respectively.

In general, the type of alkyne insertion in the transition metal-catalyzed borohydride reaction determines the *cis* configuration of the final product. However, in the case of asymmetric internal alkynes as reactants, the problem of regioselectivity always exists.^16*b*,*d*^ For example, Yun *et al.* have attempted to develop a new catalytic system for the regioselective synthesis of alkenyl boron compounds with diboron reagents.^21^ Ge *et al.* reported a cobalt-catalyzed *Z*-selective hydrosilylation of alkynes relying on catalysts Co(OAC)_2_ and pyridine-2,6-diimine ligands.^22^ Recently, transition metals have been used as catalysts for the hydroboration of alkynes, and the hydroboration of HBpin with internal asymmetric alkynes has been developed. The reaction had high enantioselectivity.^23^ Furthermore, the hydroboration of alkynes has made great progress, and numerous papers on hydroboration of alkynes have been published. Most reports indicated that the active alkyne and organic solvent (such as methanol or tetrahydrofuran) participation is essential for the reaction to proceed. In continuation of our efforts to develop organic transformations in aqueous media with numerous inherent advantages than reactions in conventional organic solvents,^24^ such as the environmental protection and harmlessness of the reaction process. Herein, we, for the first time, report the palladium-catalyzed hydroboration of unactivated alkynes in water. We explored the new method under green and mild conditions, and its catalytic system reacted well and had a wide range of substrates.

## Results and discussion

In our initial experiments, we investigated the hydroboration of 4-octynes by employing a catalytic amount of Pd(PPh_3_)_4_ in the presence of B_2_(pin)_2_. The results are summarized in Table 1. When using THF as a solvent, moderate overall yield (45%) of series of isomers was obtained (Table 1, entry 1), which was confirmed by NMR and GC-MS.^25^ Fortunately, the isomerization problem was successfully avoided when the solvent THF was simply changed into pure water, which afforded a moderate yield (58%) with only one isomer (Table 1, entry 1). When 4 eq. HOAc was mixed with H_2_O, a high yield was obtained (Table 1, entry 1). Under the same conditions, when other acids were used, the yield decreased. For example, when CF_3_CO_2_H, PTSA·H_2_O and EtCO_2_H were used, only 12%, 5% and 39% yields were obtained, respectively (Table 1, entries 5, 6 and 7). It turned out that these acids were not better than HOAc. When solvents were replaced with MeOH, EA or DMF, all yields decreased (Table 1, entry 3, 4 and 8). Among the different solvents investigated, water was the best solvent for the hydroboronation reaction of 4-octynes (Table 1, entry 2). It is important to note that the employment of Pd(0) is indispensable for the hydroboration reaction, and without it, no desired product was obtained (Table 1, entry 13). It is also noteworthy to mention that only use H_2_O or HOAc as a solvent, and the reaction using Pd(PPh_3_)_4_ proceeded sluggishly to afford the desired product in a lower yield (Table 1, entries 9 and 10). Various palladium catalysts were also investigated in the hydroboration reaction, and when using Pd(OAc)_2_ or Pd_2_(dba)_3_ as catalysts, just trace products were obtained (Table 1, entries 11 and 12). The reaction was performed at different temperatures, as shown in Table 1, entries 14 and 15, within 60 and 70 °C, just 37% and 51% yields were obtained, respectively. However, the desired product was obtained with equivalent yields at a higher temperature, such as 90 °C (Table 1, entry 16). The reaction was also performed at different reaction times, as shown in Table 1, entries 17 and 18. When the time was 3 h and 6 h, the yields were only 32% and 41%, respectively. However, when reaction time was extended, almost same yields of the desired product were obtained with longer reaction time periods of 18 h and 24 h (Table 1, entry 19 and 20). With an optimal reaction protocol in hand, numerous unactivated alkynes were examined.

**Table 1:** Optimization of the reaction conditions using 4-octyne 1 and bis(pinacolato)-diboron 2^a^

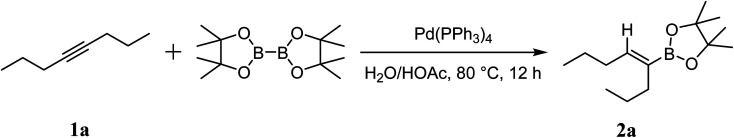					
Entry	Catalysts	Solvents	Temperature	Reaction time	Yield^b^ (%)
1	Pd(PPh_3_)_4_	THF/HOAc	80 °C	12 h	45
**2**	**Pd(PPh**_**3**_ **)**_**4**_	**H**_**2**_ **O/HOAc**	**80** °**C**	**12 h**	**58**
3	Pd(PPh_3_)_4_	MeOH/HOAc	80 °C	12 h	52
4	Pd(PPh_3_)_4_	EA/HOAc	80 °C	12 h	50
5	Pd(PPh_3_)_4_	H_2_O/CF_3_CO_2_H	80 °C	12 h	12
6	Pd(PPh_3_)_4_	H_2_O/PTSA·H_2_O	80 °C	12 h	5
7	Pd(PPh_3_)_4_	H_2_O/EtCO_2_H	80 °C	12 h	39
8	Pd(PPh_3_)_4_	DMF/HOAc	80 °C	12 h	48
9	Pd(PPh_3_)_4_	HOAc	80 °C	12 h	23
10	Pd(PPh_3_)_4_	H_2_O	80 °C	12 h	12
11	Pd(OAc)_2_	H_2_O/HOAc	80 °C	12 h	Trace
12	Pd_2_(dba)_3_	H_2_O/HOAc	80 °C	12 h	Trace
13	—	H_2_O/HOAc	80 °C	12 h	0
14	Pd(PPh_3_)_4_	H_2_O/HOAc	60 °C	12 h	37
15	Pd(PPh_3_)_4_	H_2_O/HOAc	70 °C	12 h	51
16	Pd(PPh_3_)_4_	H_2_O/HOAc	90 °C	12 h	57
17	Pd(PPh_3_)_4_	H_2_O/HOAc	80 °C	3 h	32
18	Pd(PPh_3_)_4_	H_2_O/HOAc	80 °C	6 h	41
19	Pd(PPh_3_)_4_	H_2_O/HOAc	80 °C	18 h	57
20	Pd(PPh_3_)_4_	H_2_O/HOAc	80 °C	24 h	59

a Reaction conditions: Pd catalyst (5%), solvent (1.5 mL), 1a (1 eq.), B_2_(pin)_2_ (2 eq.), HOAc (4 eq.).

b Isolated yield.

As shown in Table 2, Pd(PPh_3_)_4_ efficiently catalyzed the hydroboration reactions of various alkynes and B_2_(pin)_2_ in H_2_O at 80 °C to afford the corresponding products in moderate to good yields. Both aryl- and alkyl-substituted alkynes proceeded smoothly within the reaction time to provide the desired products in good to high yields (Table 2, entries 1 and 2). With 1-phenyl-1-butyne and 1-phenyl-1-pentyne substrates, under the optimal catalytic conditions, α-vinyl boronate could be exclusively furnished in excellent yields of 85% and 70%, respectively (Table 2, entries 5 and 6). For the activated substrates (Table 2, entries 7 and 8), the reaction proceeded smoothly with 68% and 60% yields to afford the desired products. When 1-phenyl-1-heptyne and 1-phenyl-1-propyne were used as substrates, under the optimal catalytic conditions, the desired products were provided with yields of 62% and 60%, respectively (Table 2, entries 11 and 13). For 1j, the corresponding product 2j was produced under the structure of the 1,4-bisarylation group. The reaction proceeded smoothly with a yield of 74%. It further demonstrated that this reaction had a wide range of substrates. It was gratifying to find that for the substrate 1l, the reaction delivered only one regioisomer 2l (Table 2, entries 12), which may be due to the complexation effect of the *ortho* olefin substituent with the palladium catalyst.

**Table 2:** Screening the reactivity of various alkynes in the boron addition catalyzed by Pd(PPh_3_)_4_^a^

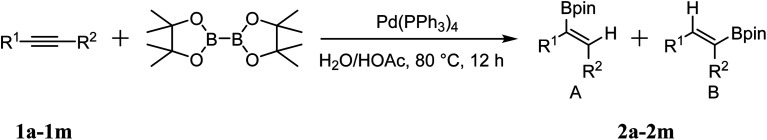				
Entry	Alkyne	Product^b^	Yield^c^ (%)	A^d^ : B
1	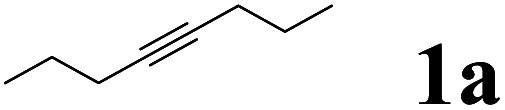	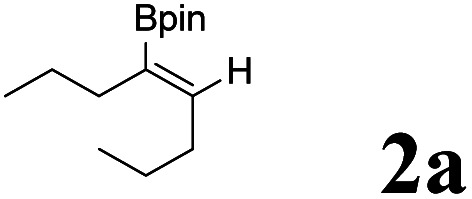	58	—
2	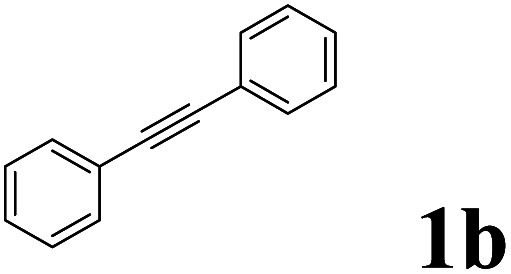	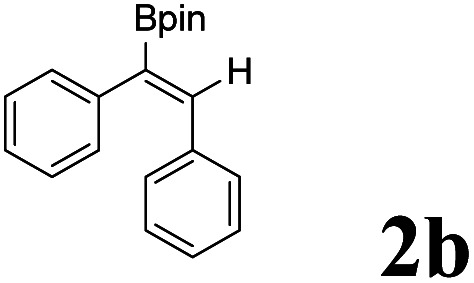	70	—
3	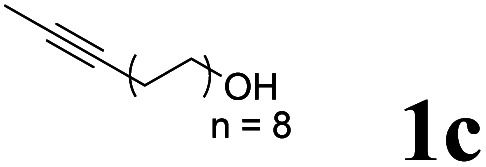	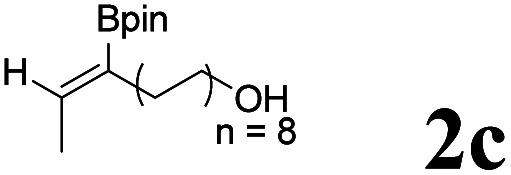	68	52 : 48
4	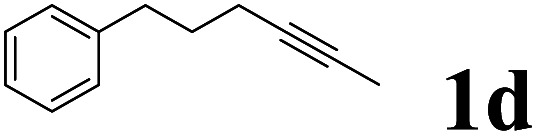	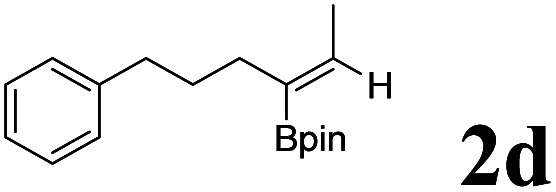	75	60 : 40
5	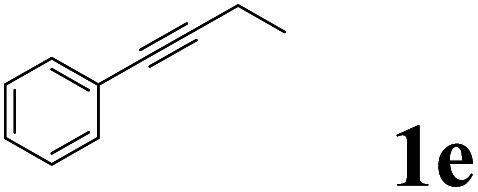	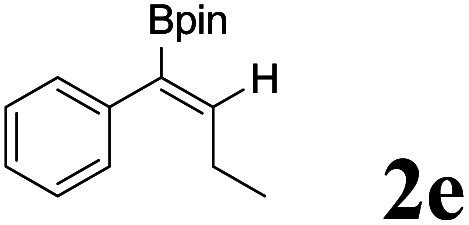	85	75 : 25
6	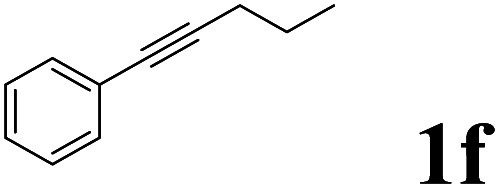	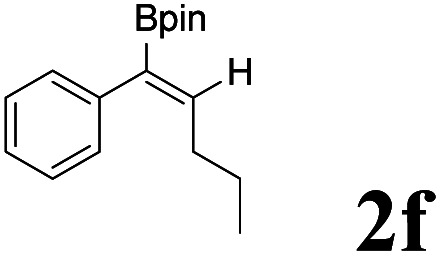	70	84 : 16
7	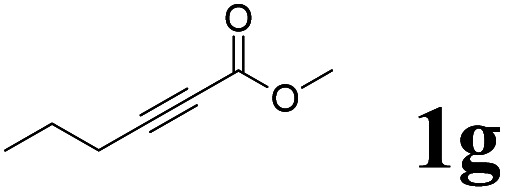	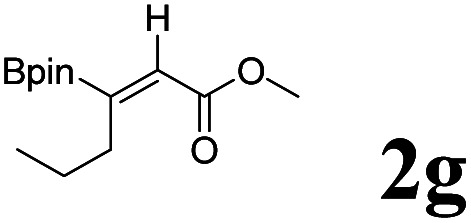	68	90 : 10
8	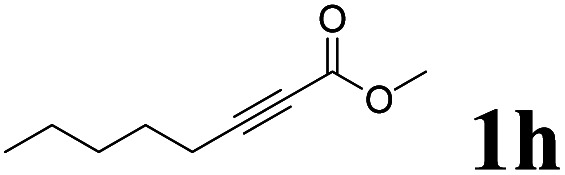	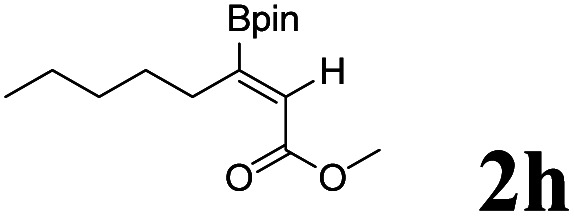	60	84 : 16
9	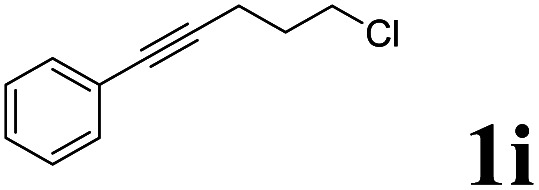	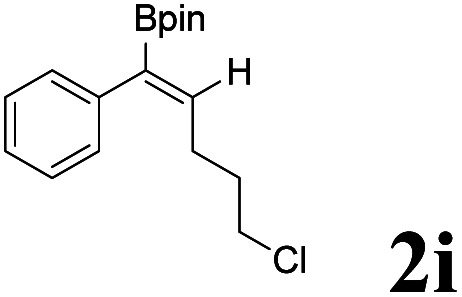	62	86 : 14
10	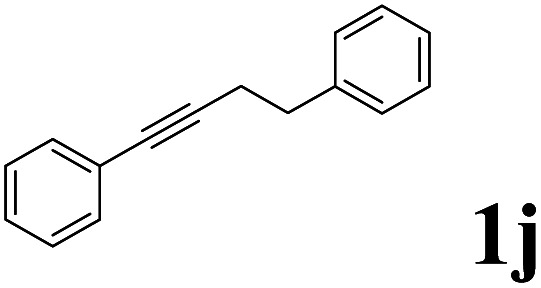	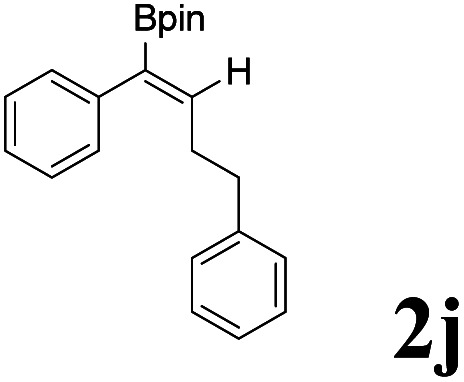	74	75 : 25
11	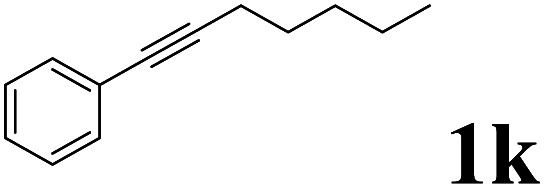	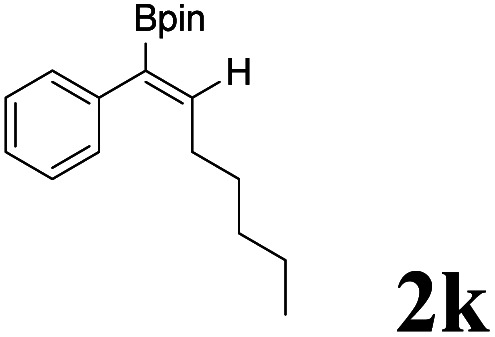	60	75 : 25
12	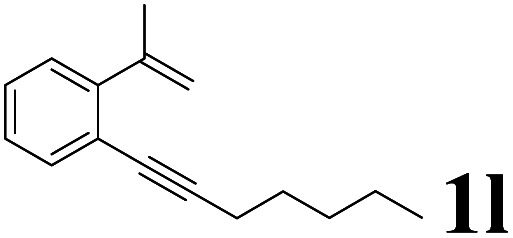	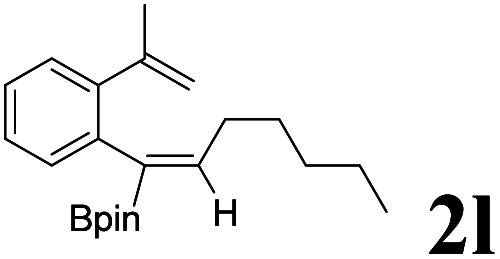	61	100 : 0
13	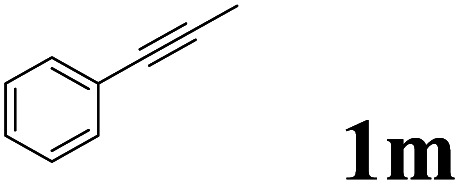	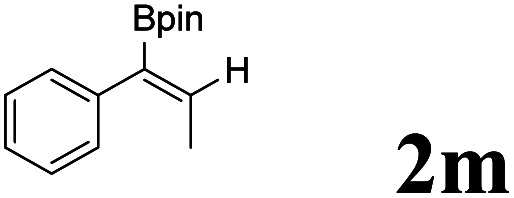	62	53 : 47

a Reaction conditions: 5 mol% Pd(PPh_3_)_4_, 1 equiv. Alkyne, 2 equiv. B_2_pin_2_, 4 equiv. HOAc in 1.5 mL H_2_O at 80 °C for 12 h.

b The drawing structure is the major regioisomer.

c Isolated yield.

d Determined by ^1^H-NMR of the crude product.

It is exciting to find that with the substrate 1l, the reaction delivered only one regioisomer 2l (Table 2, entry 12) and 2l was in the *Z* configuration. Therefore, this result can inspire us to use this type of substrate to produce the product we need, without producing other by-products, and to conduct a strongly targeted synthesis. Such a high yield product can be applied in industrial synthesis. Furthermore, functional groups such as hydroxyl, ester and halide were all tolerated in this reaction, which further proved the high compatibility of this transformation (Table 2, entries 3 and 7–9).

**Scheme 2:**
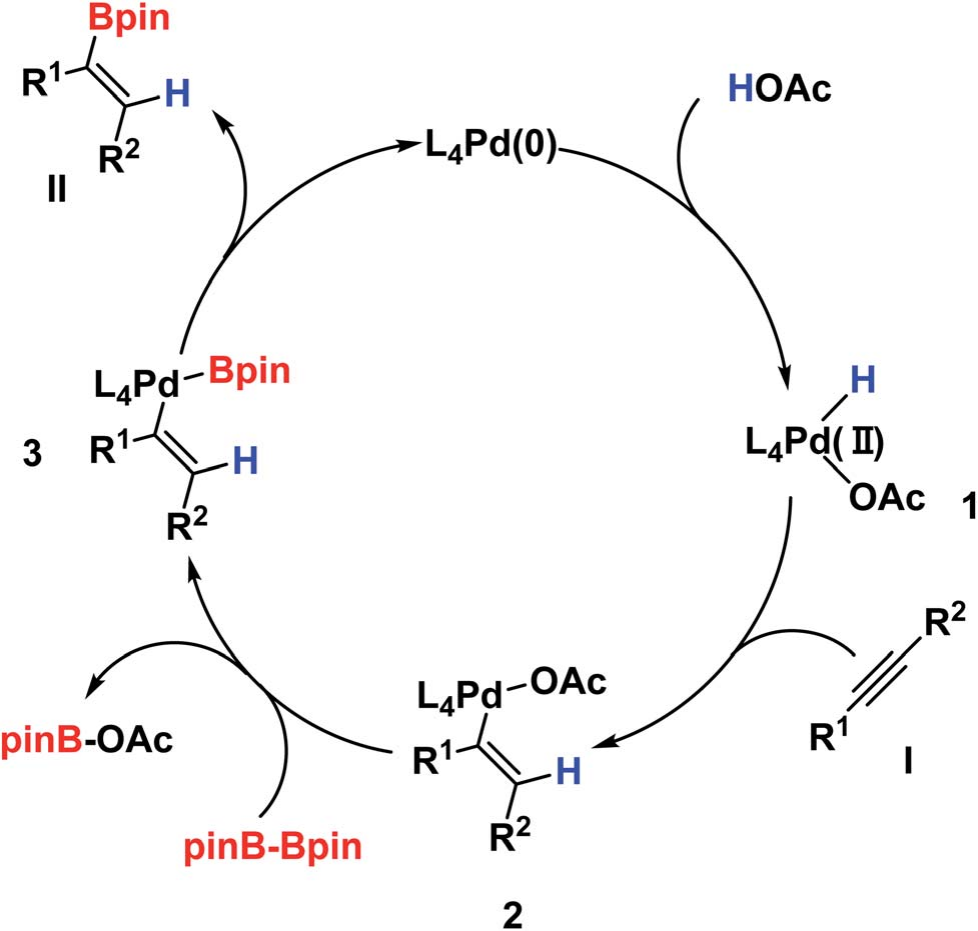
Proposed reaction mechanism.

On the basis of our experiments as well as literature precedents,^15*b*^ we proposed the mechanism, which is shown in Scheme 2. The palladium(0) complex initially delivers the palladium hydride complex (intermediate 1) after the reaction with acetic acid, and the hydrogen atom transfers from AcOH to palladium, providing the [PdH] species 1. Next, intermediate 1 subsequently produces intermediate 2 after complexation and migratory insertions with alkyne I. Then, intermediate 2 undergoes a transmetallation with B_2_pin_2_ and furnishes intermediate 3. Lastly, intermediate 3, after a reductive elimination would generate the desired product II and regenerate the palladium(0) complex to close the catalytic cycle.

## Conclusion

In summary, we have developed an efficient procedure for the hydroboration reactions with unactivated alkynes based on a palladium catalyst, which provides a route for the synthesis of α-borylated-α,β-alkenes in water. These studies suggested that H_2_O is a suitable solvent for the palladium-catalyzed hydroboration reaction. Studies are underway to extend the application of the boron derivatives in organic syntheses.

## Author contributions

MY, YY, WM and GZ executed the synthesis of the compounds and analysis of spectral data. MY, YY, YY and DL conceptualized the research work, wrote the main text. MY, YF, YW and FZ were involved in troubleshooting the synthesis and writing the ESI.† YY and DL provided the funding acquisition. All authors reviewed the manuscript and the ESI.†

## Conflicts of interest

The authors declare no conflicts of interest.

## Supplementary Material

RA-012-D1RA09136K-s001
