# Supplementary material for: Palladium-catalyzed hydroboration reaction of unactivated alkynes with bis (pinacolato) diboron in water
Source: RSC Adv. 2022 Mar 29;12(16):9815–20. doi: 10.1039/d1ra09136k (PMC8961796; doi:10.1039/d1ra09136k)

Electronic Supplementary Material (ESI) for RSC Advances.
This journal is © The Royal Society of Chemistry 2022

Palladium catalyzed hydroboration reaction of unactivated alkynes with bis(pinacolato)diboron in water

Ming Yang[a#], Yunzi Yu[a#], Wenxia Ma[a], Yuqin Feng[a], Gang Zhang[a], Yaqi Wu[a], Fanyu Zhou[a], Yongsheng Yang*[a], Dezheng Liu*[b]

[a] School of Chemistry and Engineering, Wuhan Textile University, 1 Textile Road, Wuhan, China, 430073; Hubei Key Laboratory of Biomass Fibers and Eco-dyeing & Finishing, Wuhan Textile University, Wuhan, Hubei, China, 430073. *E-mail: ysyang@wtu.edu.cn*

[b] School of Mechanical Engineering, Hubei University of Arts and Science, No. 296 Longzhong Road, Xiangyang, Hubei Province, P.R. China 41053. *E-mail: Liudezheng@126.com*

[#] These authors contributed equally to this work.

Table of contents

General methods……………………………………………………………………**S2**

Experimental procedures……………………………………………………………**S2**

Spectroscopic data of products……………………………………………………**S3**

^{1}H and ^{13}C NMR Spectra of Products…………………………………………**S8**

General methods

The alkynes, B_2pin_2 and acetic acid were purchased from Aldrich and used directly without purification. Some alkynes were synthesized according to the reported method (Zhang, Xiaoxia; Sarkar, Sampa; Larock, Richard C. *J. Org. Chem.* **2006**, *71*, 236).

The following commercial grade solvents and reagents were also used without further purification: $Pd(PPh_3)_4$ (powder, 99.9%, Aldrich chemical), HOAc (Aldrich chemical).

Deionized water was used in all reactions.

The stirrer was “Mr 3001K” type, purchased from Heidolph. It was stirred at the largest speed of 1250 rpm. The stirring bar was egg-shaped in 1.6 cm × 0.6 cm (L × Diam.).

Analytical thin layer chromatography (TLC) was performed using Merck 60 F254 precoated silica gel plate (0.2 mm thickness). Subsequent to elution, plates were visualized using UV radiation (254 nm) on Spectroline Model ENF-24061/F 254 nm. Further visualization was possible by staining with acidic solution of ceric molybdate.

Flash chromatography was performed using Merck silica gel 60 with freshly distilled solvents. Columns were typically packed as slurry and equilibrated with the appropriate solvent system prior to use.

Infrared spectra were recorded on a Bio-Rad FTS 165 FTIR spectrometer.

High Resolution Mass Spectra (HRMS) were obtained using Waters Q-Tof Permies Mass Spectrometer.

Proton nuclear magnetic resonance spectra (^1H NMR) were recorded on a Bruker Avance DPX 300, Bruker AMX 400 spectrophotometer and Bruker AMX 500 spectrophotometer ($CDCl_3$ as solvent). Chemical shifts for ^1H NMR spectra are reported as δ in units of parts per million (ppm) downfield from $SiMe_4$ (δ 0.0) and relative to the signal of chloroform-*d* (δ 7.2600, singlet). Multiplicities were given as: s (singlet); d (doublet); t (triplet); q (quartet); or m (multiplets). The number of protons (n) for a given resonance is indicated by nH. Coupling constants are reported as a J value in Hz. Carbon nuclear magnetic resonance spectra (^{13}C NMR) are reported as δ in units of parts per million (ppm) downfield from $SiMe_4$ (δ 0.0) and relative to the signal of chloroform-*d* (δ 77.0, triplet). Diastereoselectivity was determined by isolation and/or ^1H NMR analysis.

Experimental procedures

General procedure for the hydroboration reaction

To a 8 mL sample vial was added tetrakis(triphenylphosphine)palladium(0) (0.01 mmol), alkyne (0.2 mmol), B_2pin_2 (0.4 mmol), water (1.5 mL) and acetic acid (0.8 mmol) sequentially, then it was stirred vigorously at 80 °C for 12 hours. After the reaction, 2 mL H_2O was added, and then it was extracted using diethyl ether (5 mL × 4), washed with brine, dried over anhydrous sodium sulfate, filtered and evaporated solvent under *vacuo* to give the residue. It was subjected to silica gel column chromatography using ethyl acetate and hexane as eluent to afford the desired product.

Spectroscopic data of products

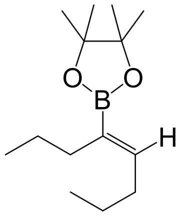

(*Z*)-4,4,5,5-tetramethyl-2-(oct-4-en-4-yl)-1,3,2-dioxaborolane (2a): Colorless oil; Yield: 58%; R_f = 0.55 (Ethyl acetate/Hexane 1:10); FTIR (KBr, neat): ν 1628 cm^{-1} (C=C); ^1H NMR (400 MHz, $CDCl_3$): δ 0.88 (t, J = 7.33 Hz, 3H), 0.92 (t, J = 7.36 Hz, 3H), 1.25 (s, 12H), 1.33-1.44 (m, 4H), 2.08-2.14 (m, 4H), 6.30 (t, J = 7.08 Hz, 1H) ppm; ^{13}C NMR (100 MHz, $CDCl_3$): δ 146.0, 82.9, 30.6, 30.6, 24.7, 23.3, 22.4, 14.1, 14.0 ppm; HRMS (ESI, m/z): Calcd. For $C_{14}H_{28}BO_2$: 239.2182, found $[M+H]^+$: 239.2189.

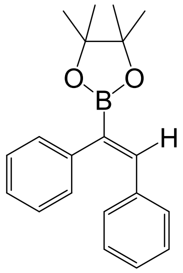

(*Z*)-2-(1,2-diphenylvinyl)-4,4,5,5-tetramethyl-1,3,2-dioxaborolane (2b): White solid; Yield: 70%; R_f = 0.55 (Ethyl acetate/Hexane 1:10); FTIR (KBr, neat): ν 1636 cm^{-1} (C=C); ^1H NMR (300 MHz, $CDCl_3$): δ 1.31 (s, 12H), 7.03-7.29 (m, 10H), 7.36 (s, 1H) ppm; ^{13}C NMR (100 MHz, $CDCl_3$): δ 143.1, 140.4, 137.0, 129.9, 128.8, 128.2, 127.8, 127.5, 126.2, 83.8, 24.8 ppm; HRMS (ESI, m/z): Calcd. For $C_{20}H_{24}BO_2$: 307.1869, found $[M+H]^+$: 307.1869.

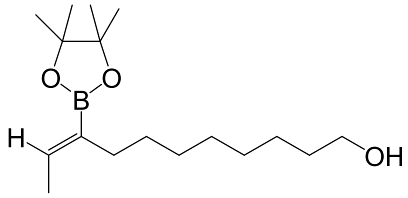

(*Z*)-9-(4,4,5,5-tetramethyl-1,3,2-dioxaborolan-2-yl)undec-9-en-1-ol (2c): Light yellow oil; Yield: 68%; R_f = 0.43 (Ethyl acetate/Hexane 1:2); FTIR (KBr, neat): ν 1632 cm^{-1} (C=C); ^1H NMR (500 MHz, $CDCl_3$): δ 1.26 (s, 12H), 1.27-1.39 (m, 11H), 1.53-1.59 (m, 2H), 1.67-1.71 (m, 3H), 2.09-2.13 (m, 2H), 3.63 (t, J = 6.60 Hz, 2H), 6.30-6.42 (m, 1H) ppm; ^{13}C NMR (125 MHz, $CDCl_3$): δ 146.6, 140.1, 83.0, 82.9, 63.1, 63.0, 32.8, 32.8, 29.8, 29.5, 29.4, 29.4, 29.4, 29.3, 28.8, 28.6, 28.0, 25.7, 25.7, 24.8, 24.7, 14.2, 13.8 ppm; HRMS (ESI, m/z): Calcd. For $C_{17}H_{34}BO_3$: 297.2601, found $[M+H]^+$: 297.2608.

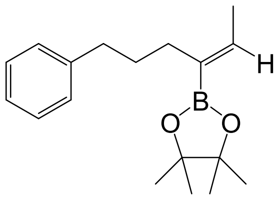

(*Z*)-4,4,5,5-tetramethyl-2-(6-phenylhex-2-en-3-yl)-1,3,2-dioxaborolane (2d): Light yellow oil; Yield: 75%; R_f = 0.56 (Ethyl acetate/Hexane 1:10); FTIR (KBr, neat): ν 1632 cm^{-1} (C=C); ^{1}H NMR (400 MHz, $CDCl_3$): δ 1.27 (s, 12H), 1.67-1.78 (m, 5H), 2.15-2.23 (m, 2H), 2.59-2.65 (m, 2H), 6.33-6.46 (m, 1H), 7.17-7.28 (m, 5H) ppm; ^{13}C NMR (100 MHz, $CDCl_3$): δ 146.0, 143.0, 142.4, 140.6, 128.4, 128.4, 128.2, 128.2, 125.6, 125.5, 83.1, 83.0, 35.7, 35.6, 31.5, 30.4, 28.2, 27.9, 24.8, 24.7, 14.2, 13.9 ppm; HRMS (ESI, m/z): Calcd. For $C_{18}H_{28}BO_2$: 287.2182, found [M+H]$^+$: 287.2185.

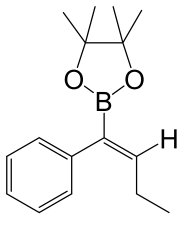

(*Z*)-4,4,5,5-tetramethyl-2-(1-phenylbut-1-enyl)-1,3,2-dioxaborolane (2e): Light yellow oil; Yield: 85%; R_f = 0.59 (Ethyl acetate/Hexane 1:10); FTIR (KBr, neat): ν 1620 cm^{-1} (C=C); ^{1}H NMR (400 MHz, $CDCl_3$): (major isomer) δ 1.00 (t, J = 7.53 Hz, 3H), 1.27 (s, 12H), 2.12-2.20 (m, 2H), 6.57 (t, J = 7.29 Hz, 1H), 7.13-7.33 (m, 5H) ppm; ^{13}C NMR (100 MHz, $CDCl_3$): δ 149.8, 140.2, 128.9, 127.7, 125.8, 83.4, 24.7, 23.2, 13.8 ppm; HRMS (ESI, m/z): Calcd. For $C_{16}H_{24}BO_2$: 259.1869, found [M+H]$^+$: 259.1865.

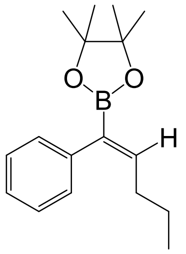

(*Z*)-4,4,5,5-tetramethyl-2-(1-phenylpent-1-enyl)-1,3,2-dioxaborolane (2f): Light yellow oil; Yield: 70%; R_f = 0.31 (Ethyl acetate/Hexane 1:10); FTIR (KBr, neat): ν 1616 cm^{-1} (C=C); ^{1}H NMR (300 MHz, $CDCl_3$): (major isomer) δ 0.87 (t, J = 7.39 Hz, 3H), 1.27 (s, 12H), 1.36-1.49 (m, 2H), 2.12 (q, J = 7.50 Hz, 2H), 6.58 (t, J = 7.26 Hz, 1H), 7.12-7.33 (m, 5H) ppm; ^{13}C NMR (75 MHz, $CDCl_3$): δ 148.3, 140.3, 129.0, 127.7, 125.8, 83.4, 32.0, 24.8, 22.6, 14.0 ppm; HRMS (ESI, m/z): Calcd. For $C_{17}H_{26}BO_2$: 273.2026, found [M+H]$^+$: 273.2026.

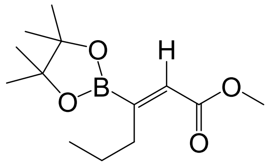

(*Z*)-methyl 3-(4,4,5,5-tetramethyl-1,3,2-dioxaborolan-2-yl)hex-2-enoate (2g): Light yellow oil; Yield: 68%; R_f = 0.64 (Ethyl acetate/Hexane 1:2); FTIR (KBr, neat): v 1636 cm^{-1} (C=C), 1715 cm^{-1} (C=O); ^1H NMR (400 MHz, $CDCl_3$): (major isomer) δ 0.93 (t, J = 7.40 Hz, 3H), 1.27 (s, 12H), 1.39-1.52 (m, 2H), 2.65 (t, J = 7.65 Hz, 2H), 3.71 (s, 3H), 6.42 (s, 1H) ppm; ^{13}C NMR (100 MHz, $CDCl_3$): δ 166.4, 129.4, 84.0, 51.1, 31.9, 24.7, 22.8, 14.1 ppm; HRMS (ESI, m/z): Calcd. For $C_{13}H_{24}BO_4$: 255.1768, found $[M+H]^+$: 255.1772.

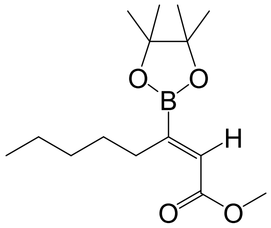

(*Z*)-methyl 3-(4,4,5,5-tetramethyl-1,3,2-dioxaborolan-2-yl)oct-2-enoate (2h): Light yellow oil; Yield: 60%; R_f = 0.22 (Ethyl acetate/Hexane 1:10); FTIR (KBr, neat): v 1633 cm^{-1} (C=C), 1720 cm^{-1} (C=O); ^1H NMR (300 MHz, $CDCl_3$): (major isomer) δ 0.88 (t, J = 6.70 Hz, 3H), 1.27 (s, 12H), 1.30-1.35 (m, 4H), 1.38-1.48 (m, 2H), 2.66 (t, J = 7.50 Hz, 2H), 3.71 (s, 3H), 6.40 (s, 1H) ppm; ^{13}C NMR (75 MHz, $CDCl_3$): δ 166.4, 129.2, 84.0, 51.0, 31.9, 30.0, 29.2, 24.7, 22.5, 14.0 ppm; HRMS (ESI, m/z): Calcd. For $C_{15}H_{28}BO_4$: 283.2081, found $[M+H]^+$: 283.2084.

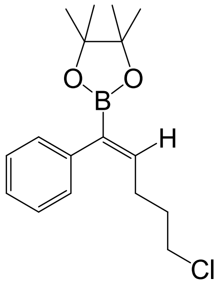

(Z)-2-(5-chloro-2-methyl-1-phenylpent-1-enyl)-4,4,5,5-tetramethyl-1,3,2-dioxaborolane (2i): White solid; Yield: 62%; R_f = 0.24 (Ethyl acetate/Hexane 1:10); FTIR (KBr, neat): v 1622 cm^{-1} (C=C); ^1H NMR (500 MHz, $CDCl_3$): (major isomer) δ 1.27 (s, 12H), 1.84-1.90 (m, 2H), 2.27-2.32 (m, 2H), 3.47 (t, J = 6.80 Hz, 2H), 6.53 (t, J = 7.19 Hz, 1H), 7.12-7.38 (m, 5H) ppm; ^{13}C NMR (125 MHz, $CDCl_3$): δ 145.8, 139.8, 128.8, 127.8, 126.1, 83.6, 44.5, 32.2, 27.2, 24.7 ppm; HRMS (ESI, m/z): Calcd. For $C_{17}H_{25}BO_2Cl$: 307.1636, found $[M+H]^+$: 307.1644.

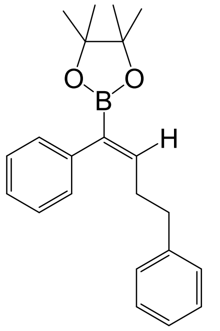

(*Z*)-2-(1,4-diphenylbut-1-enyl)-4,4,5,5-tetramethyl-1,3,2-dioxaborolane (2j): White solid; Yield: 74%; R_f = 0.21 (Ethyl acetate/Hexane 1:10); FTIR (KBr, neat): v 1616 cm^{-1} (C=C); ^1H

NMR (500 MHz, $CDCl_3$): (major isomer) δ 1.27 (s, 12H), 2.43-2.48 (m, 2H), 2.70-2.73 (m, 2H), 6.64 (t, J = 7.20 Hz, 1H), 7.07-7.32 (m, 10H) ppm; ^{13}C NMR (125 MHz, $CDCl_3$): δ 147.0, 141.8, 139.9, 128.8, 128.3, 128.3, 127.8, 125.9, 125.8, 83.5, 35.6, 31.9, 24.7 ppm; HRMS (ESI, m/z): Calcd. For $C_{22}H_{28}BO_2$: 335.2182, found $[M+H]^+$: 335.2193.

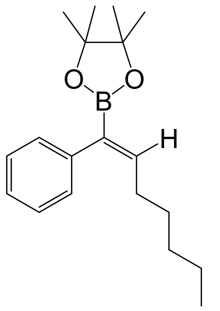

(*Z*)-4,4,5,5-tetramethyl-2-(1-phenylhept-1-enyl)-1,3,2-dioxaborolane (2k): light yellow solid; Yield: 60%; R_f = 0.37 (Ethyl acetate/Hexane 1:10); FTIR (KBr, neat): ν 1616 cm^{-1} (C=C); ^1H NMR (500 MHz, $CDCl_3$): δ 0.84 (t, J = 6.84 Hz, 3H), 1.22-1.25 (m, 4H), 1.27 (s, 12H), 1.37-1.43 (m, 2H), 2.13 (q, J = 7.65 Hz, 2H), 6.58 (t, J = 7.25 Hz, 1H), 7.13-7.33 (m, 5H) ppm; ^{13}C NMR (125 MHz, $CDCl_3$): δ 148.5, 140.3, 128.9, 127.7, 125.8, 83.4, 31.6, 29.9, 29.0, 24.7, 22.5, 14.0 ppm; HRMS (ESI, m/z): Calcd. For $C_{19}H_{30}BO_2$: 301.2339, found $[M+H]^+$: 301.2350.

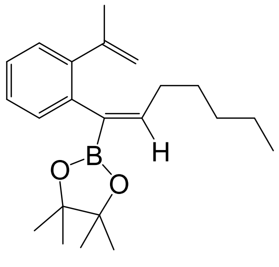

(*Z*)-4,4,5,5-tetramethyl-2-(1-(2-(prop-1-en-2-yl)phenyl)hept-1-enyl)-1,3,2-dioxaborolane (2l): White solid; Yield: 61%; R_f = 0.40 (Ethyl acetate/Hexane 1:10); FTIR (KBr, neat): ν 1618 cm^{-1} (C=C); ^1H NMR (500 MHz, $CDCl_3$): δ 0.84 (t, J = 6.90 Hz, 3H), 1.22 (s, 12H), 1.22-1.32 (m, 4H), 1.35-1.41 (m, 2H), 1.99-2.07 (m, 5H), 4.86-4.87 (m, 1H), 5.09-5.10 (m, 1H), 6.49 (t, J = 7.15 Hz, 1H), 6.99-7.01 (m, 1H), 7.18-7.23 (m, 3H) ppm; ^{13}C NMR (100 MHz, $CDCl_3$): δ 147.6, 145.2, 142.2, 138.3, 129.8, 127.3, 126.3, 126.0, 116.1, 83.2, 31.7, 30.2, 28.7, 24.7, 24.1, 22.5, 14.0 ppm; HRMS (ESI, m/z): Calcd. For $C_{22}H_{34}BO_2$: 341.2652, found $[M+H]^+$: 341.2661.

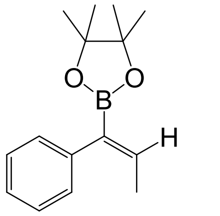

(*Z*)-4,4,5,5-tetramethyl-2-(1-phenylprop-1-enyl)-1,3,2-dioxaborolane (2m): Colorless oil; Yield: 62%; R_f = 0.44 (Ethyl acetate/Hexane 1:10); FTIR (KBr, neat): ν 1622 cm^{-1} (C=C); ^1H NMR (400 MHz, $CDCl_3$): (major isomer) δ 1.31 (s, 12H), 1.99 (d, J = 1.56 Hz, 3H), 7.30-7.39 (m,

6H) ppm; ^{13}C NMR (100 MHz, $CDCl_3$): (two isomers) δ 142.7, 142.3, 139.7, 137.9, 129.4, 129.1, 128.0, 127.7, 127.1, 125.8, 83.5, 83.4, 24.8, 24.7, 16.0, 15.9 ppm; HRMS (ESI, m/z): Calcd. For $C_{15}H_{22}BO_2$: 245.1713, found $[M+H]^+$: 245.1716.

^{1}H and ^{13}C NMR Spectra of Products

2a ^{1}H and ^{13}C NMR Spectra

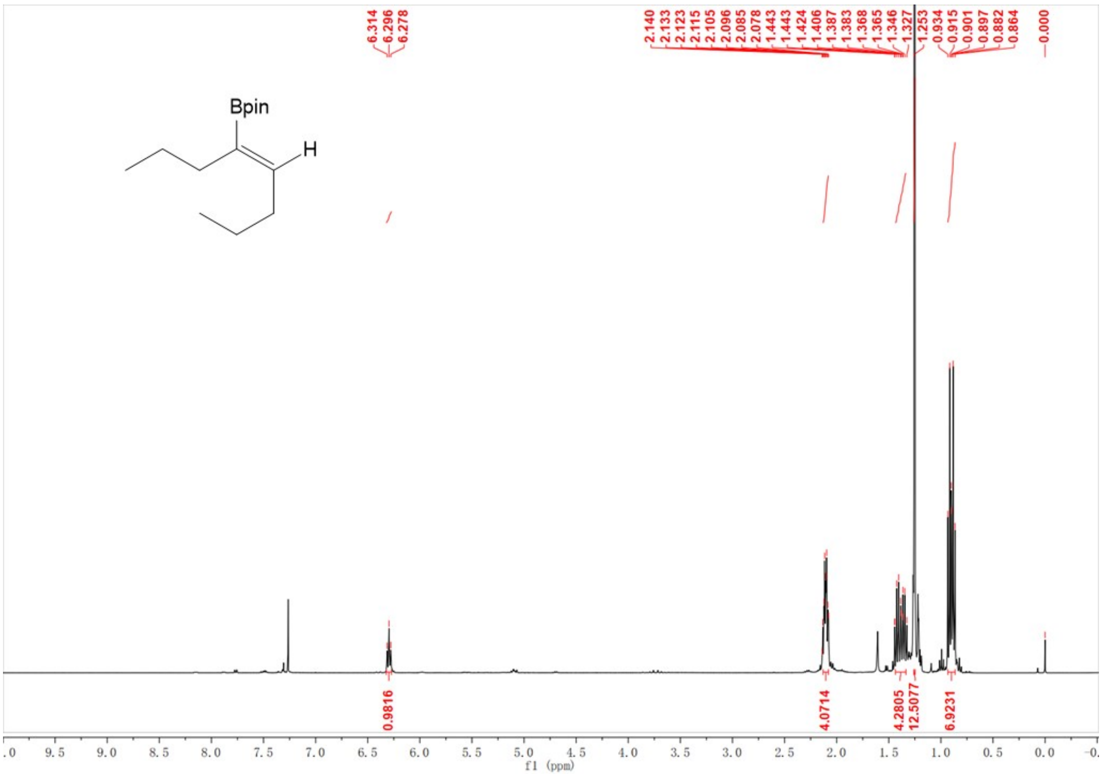

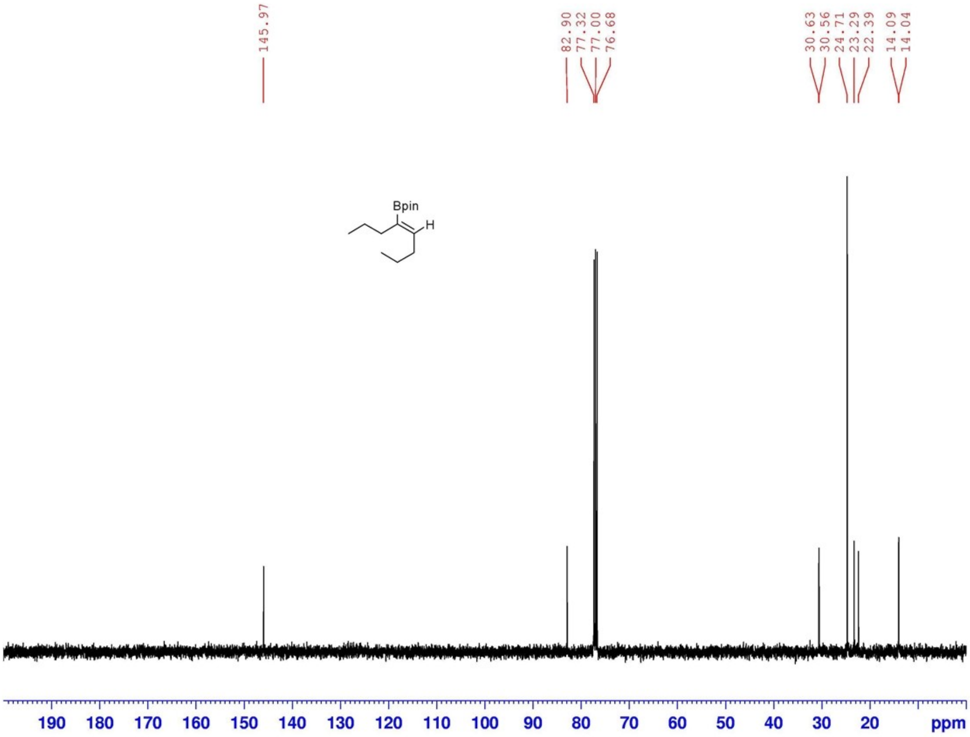

2b ^{1}H and ^{13}C NMR Spectra

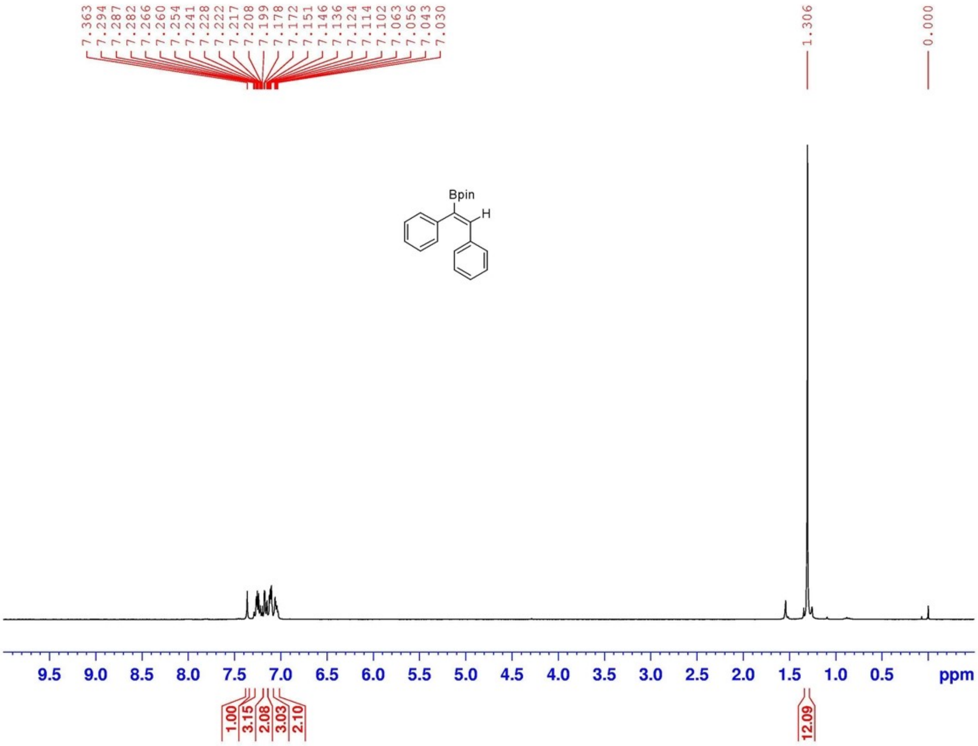

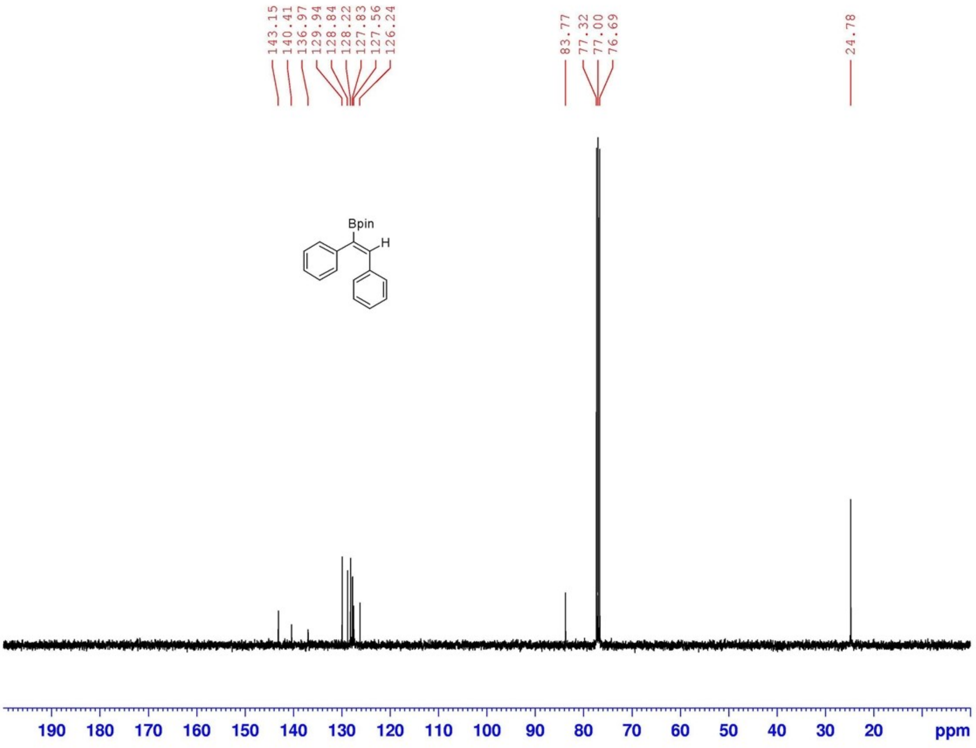

2c ^{1}H and ^{13}C NMR Spectra (The NMR spectra contain isomers)

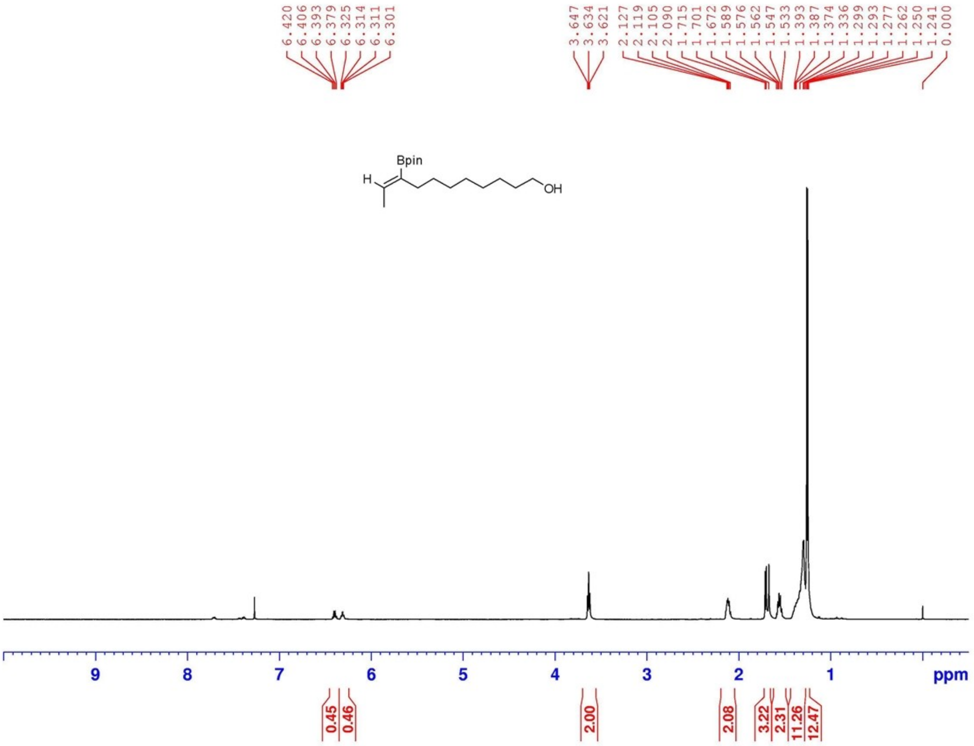

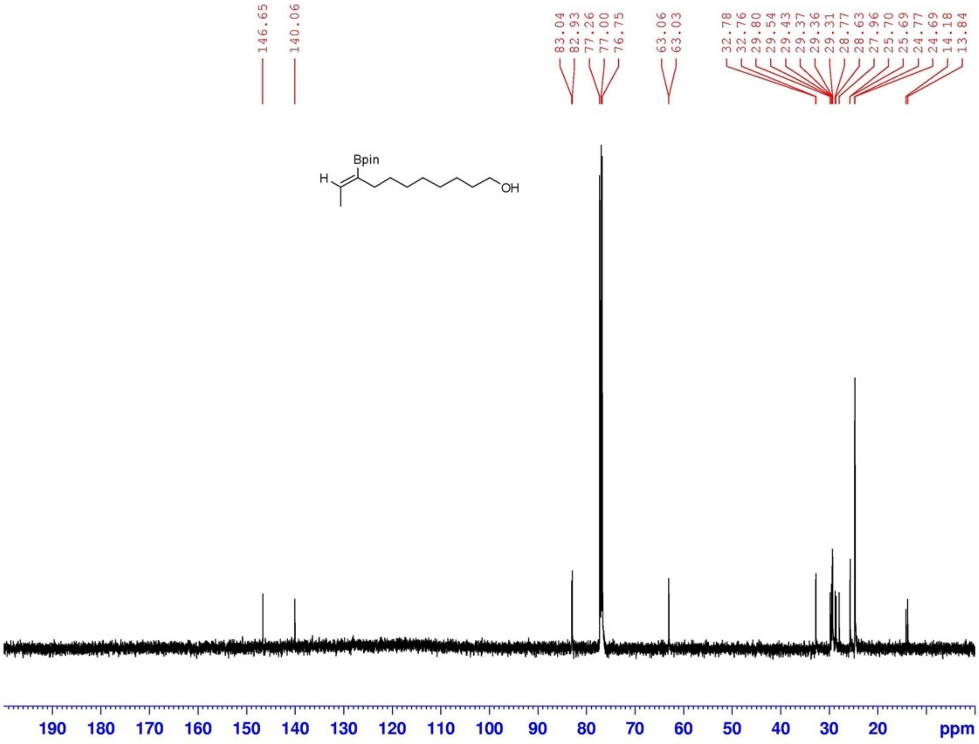

2d ^{1}H and ^{13}C NMR Spectra (The NMR spectra contain isomers)

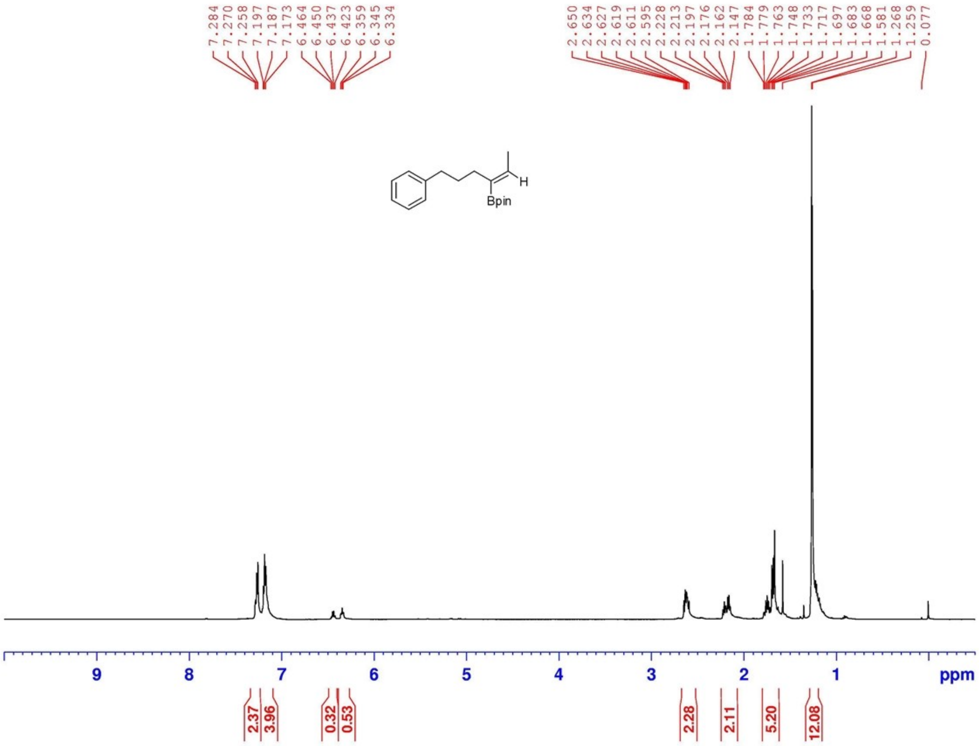

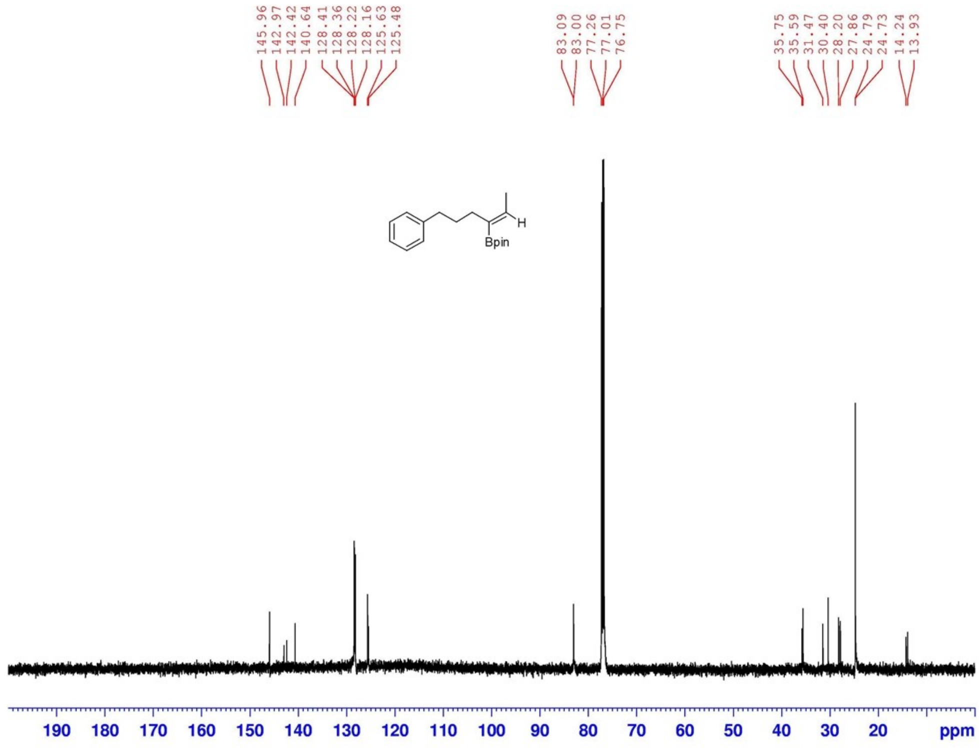

2e ^{1}H and ^{13}C NMR Spectra

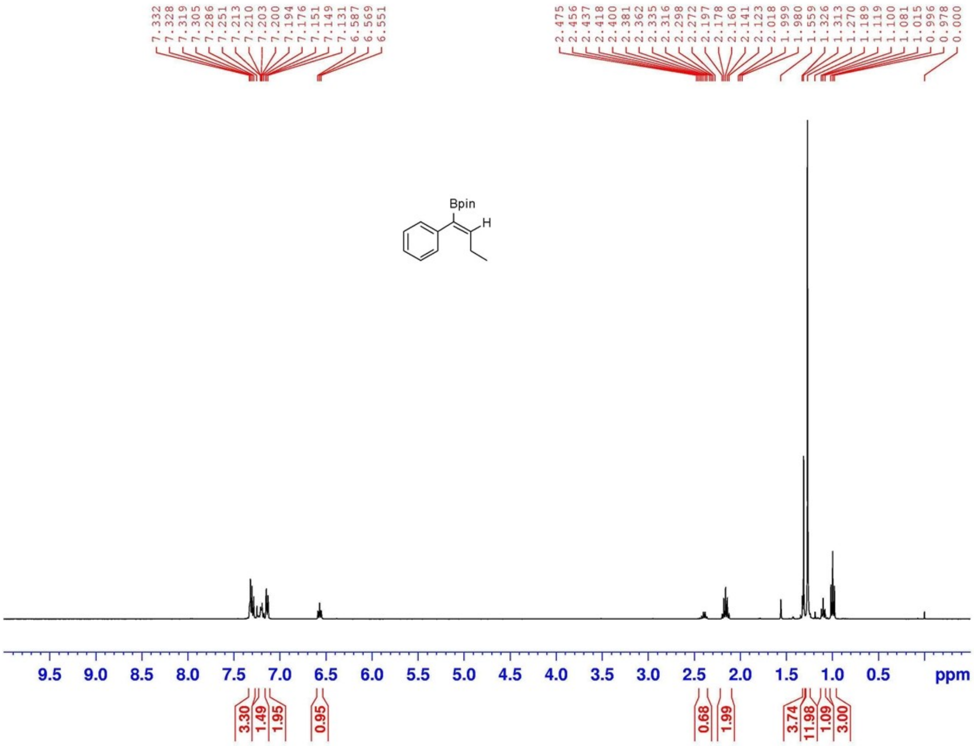

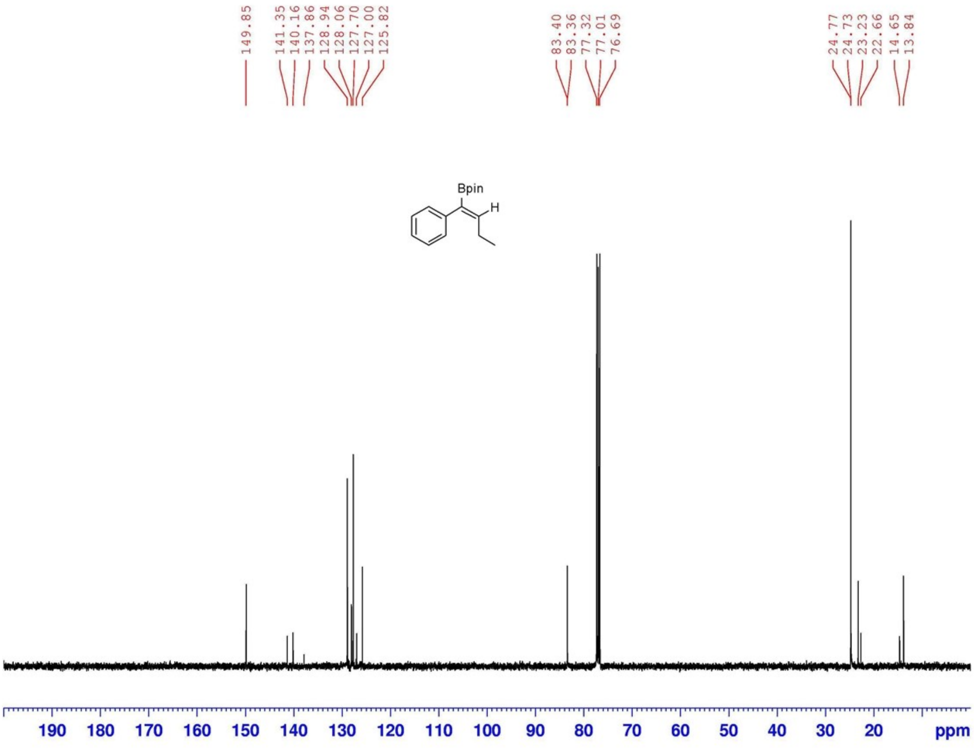

2f ^{1}H and ^{13}C NMR Spectra

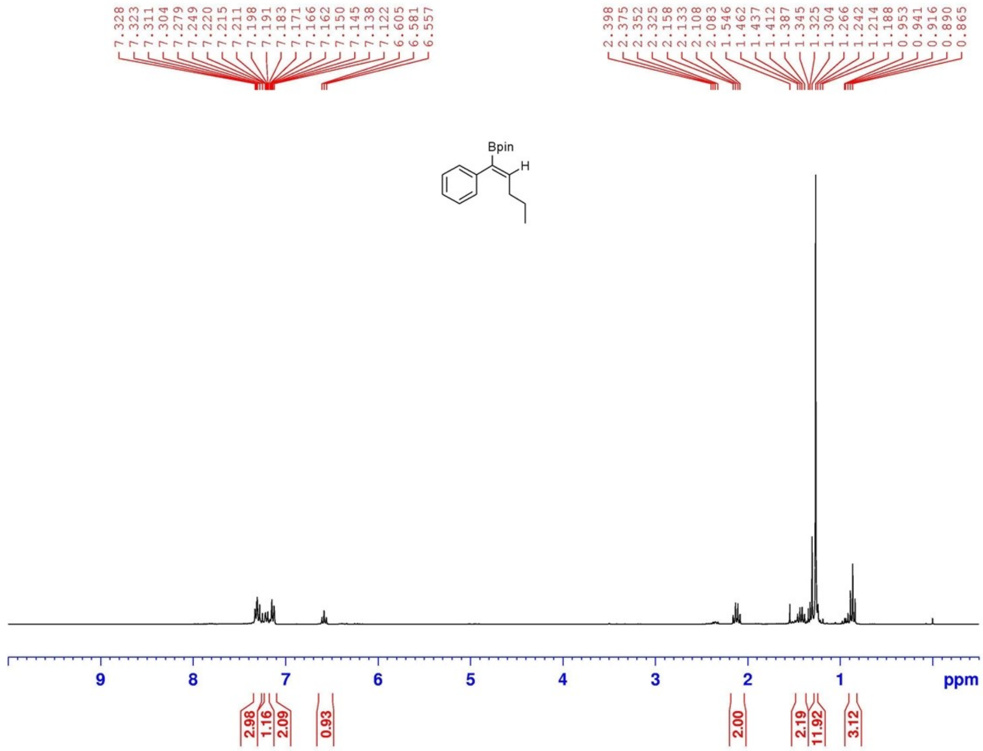

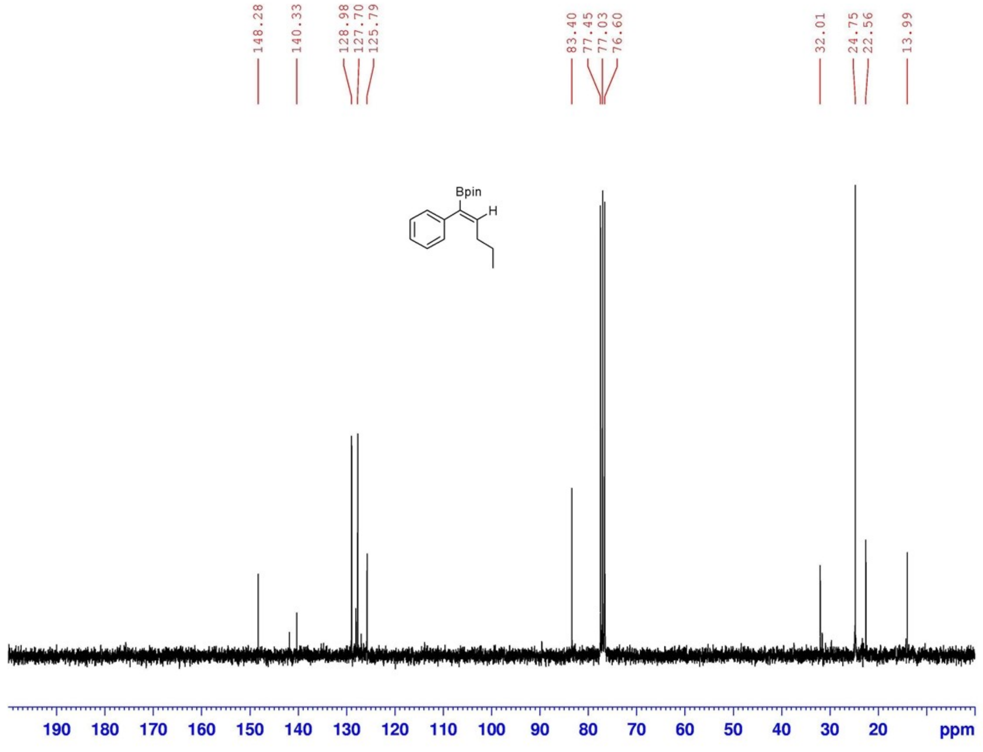

2g ^{1}H and ^{13}C NMR Spectra，NOE

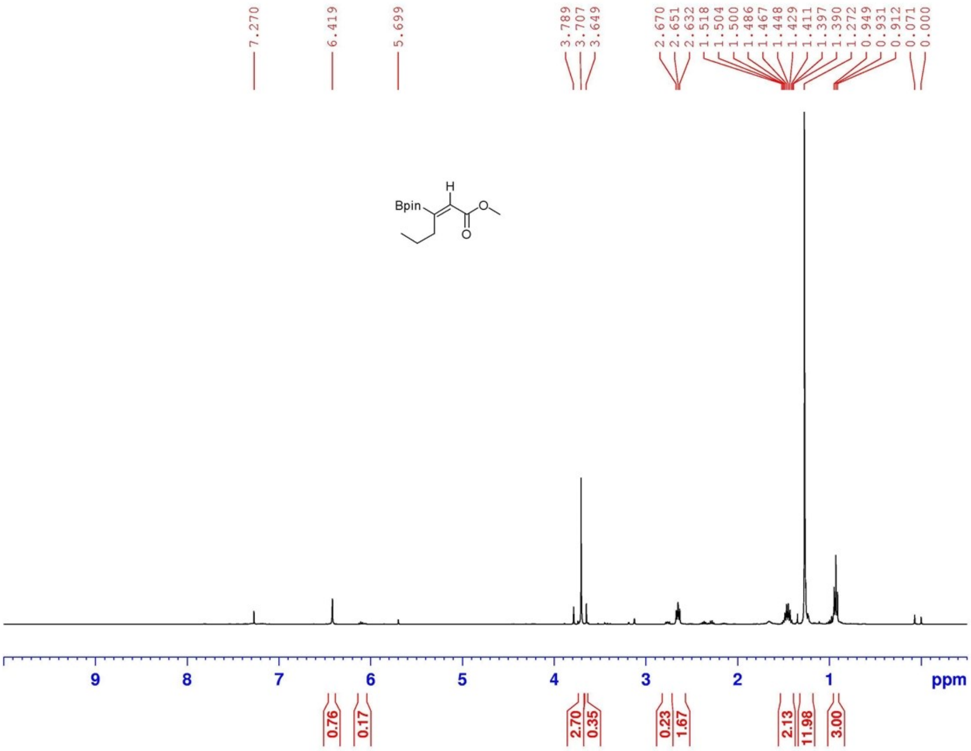

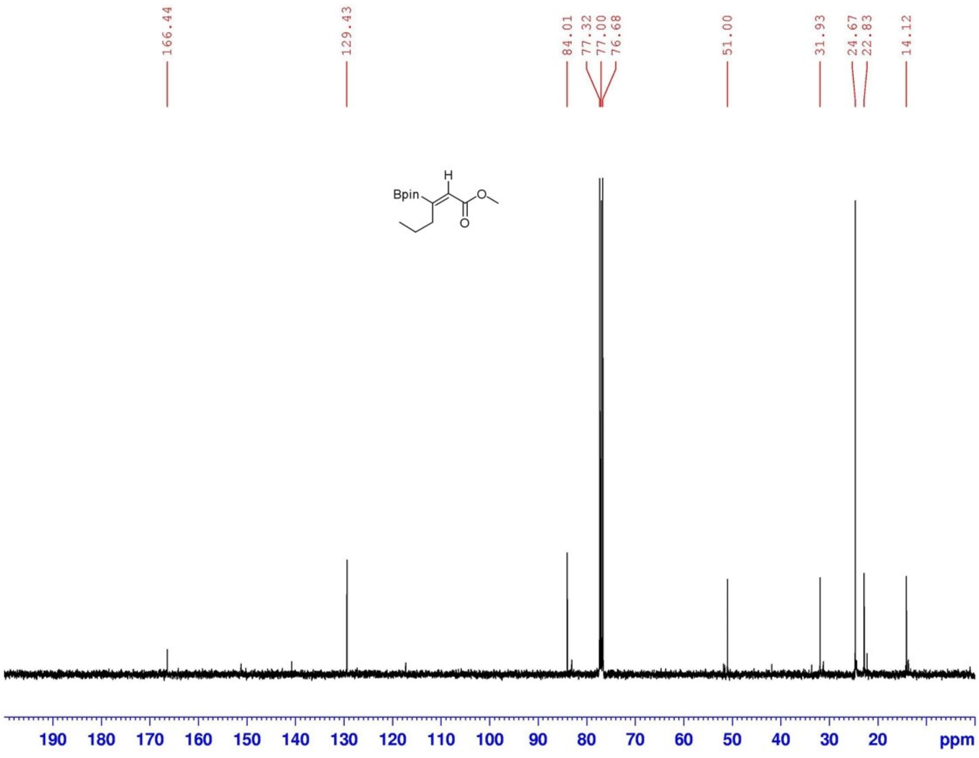

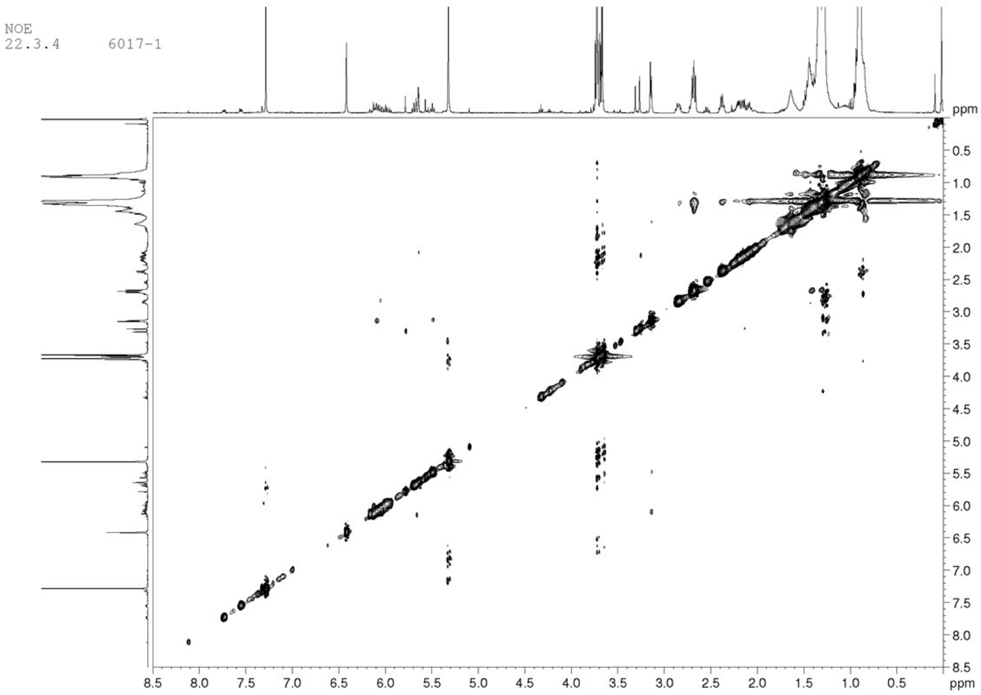

NOE
22.3.4 6017-1
ppm

2h ^{1}H and ^{13}C NMR Spectra (The NMR spectra contain isomers)

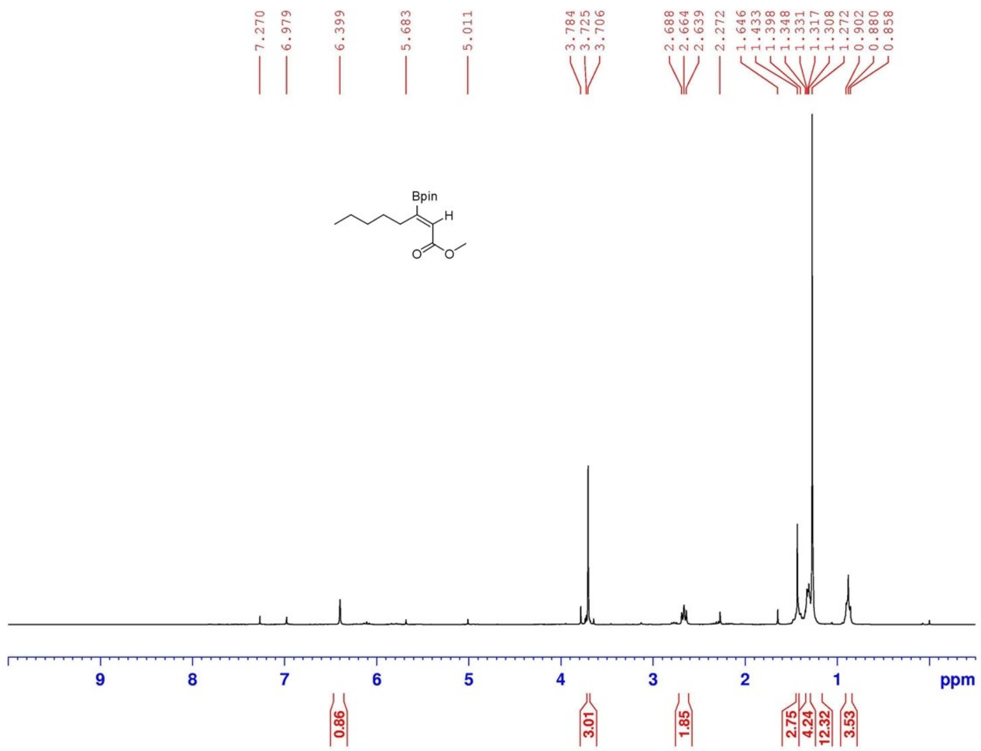

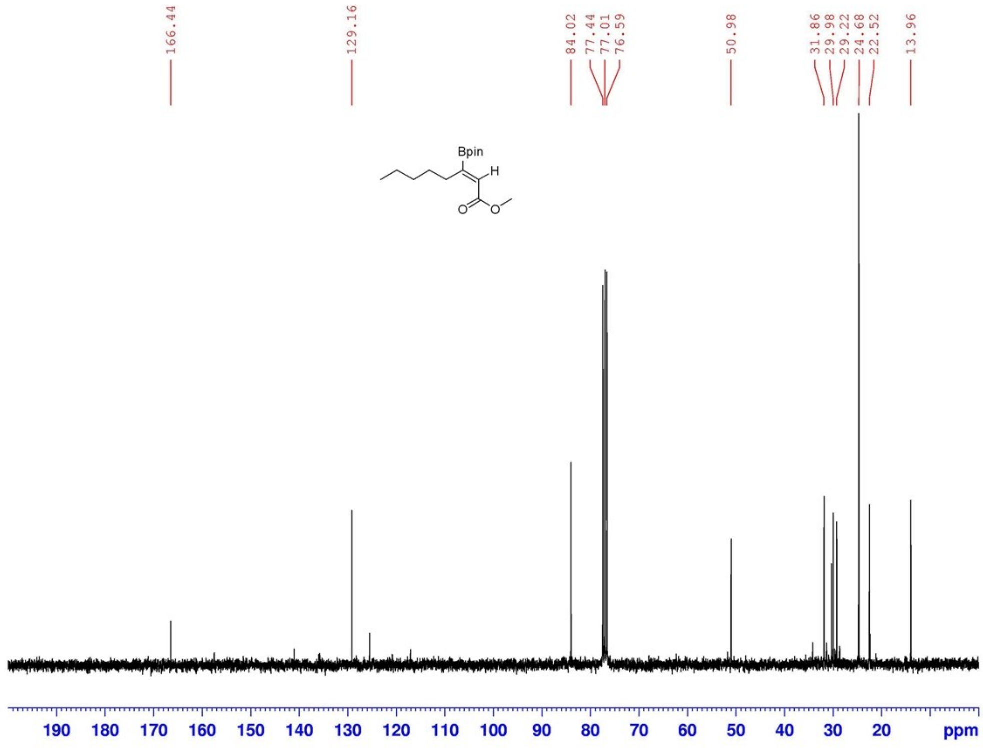

2i ^{1}H and ^{13}C NMR Spectra

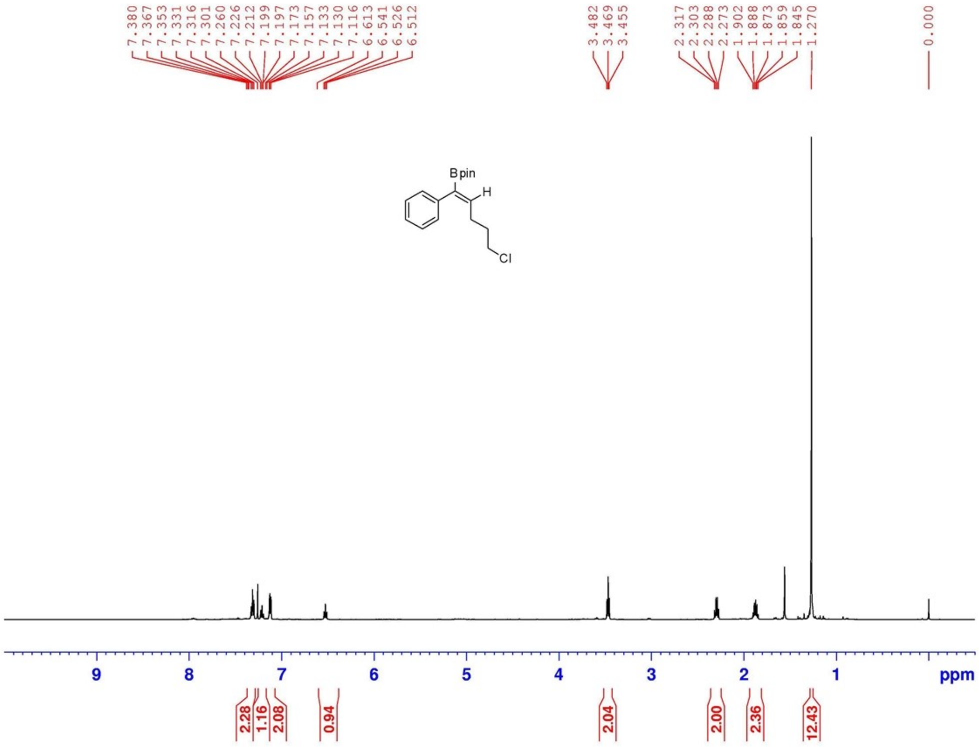

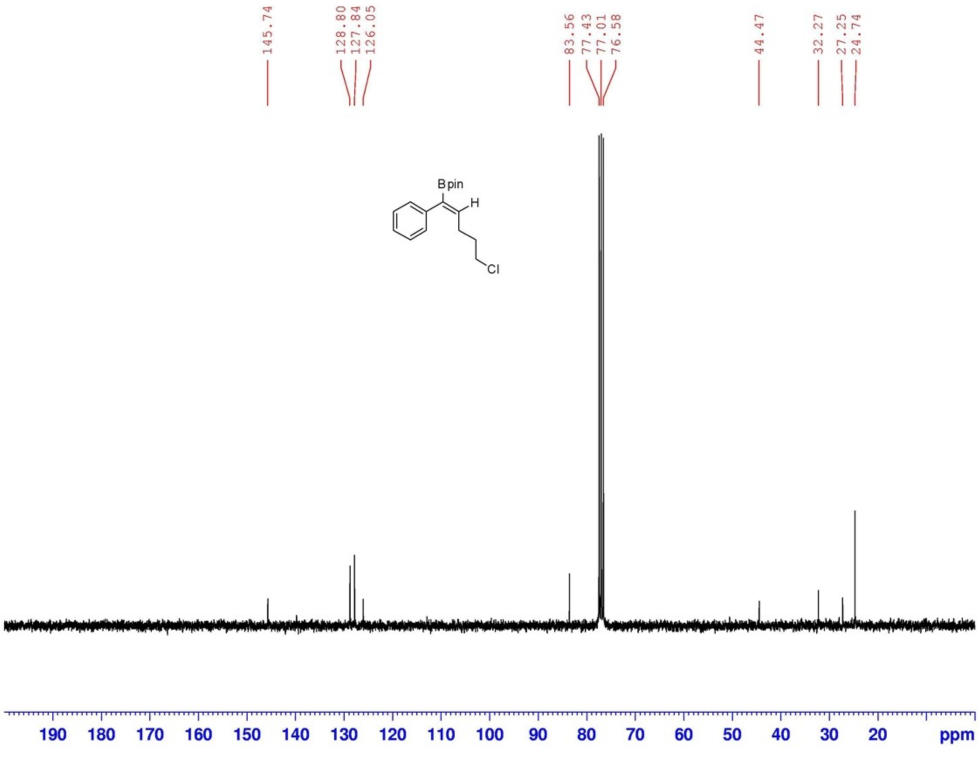

2j ^{1}H and ^{13}C NMR Spectra

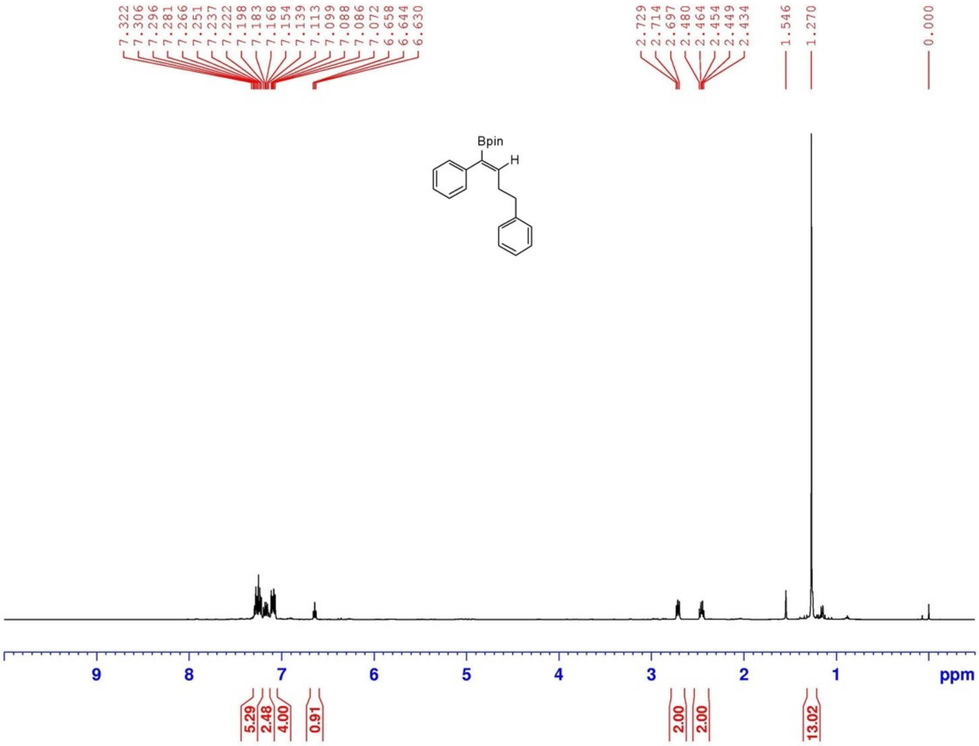

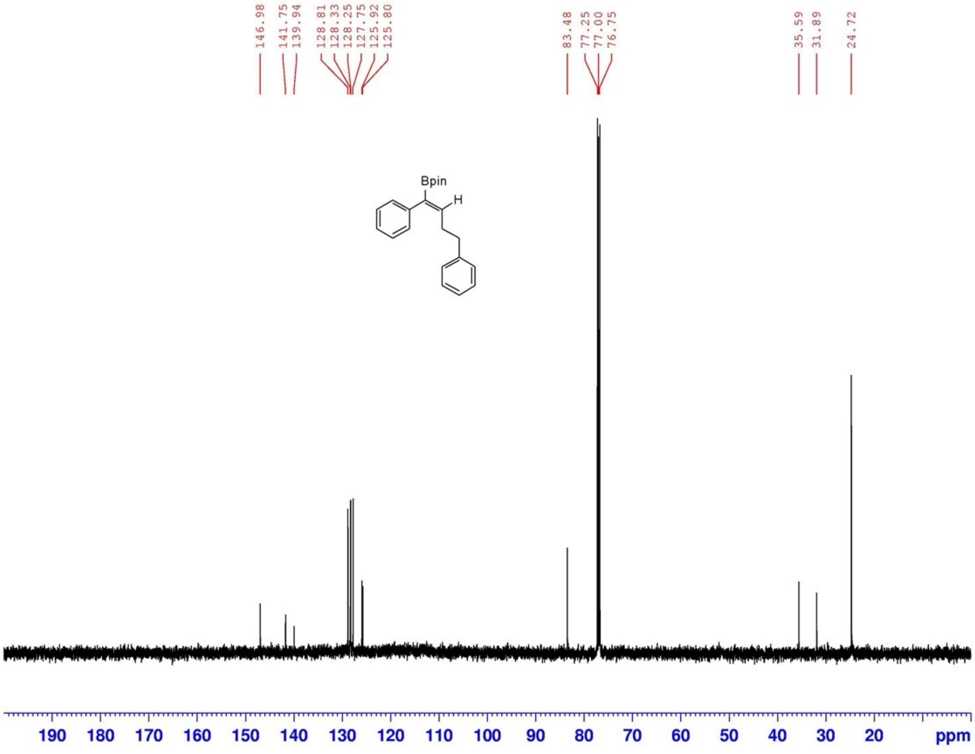

2k ^{1}H and ^{13}C NMR Spectra (The NMR spectra contain isomers)

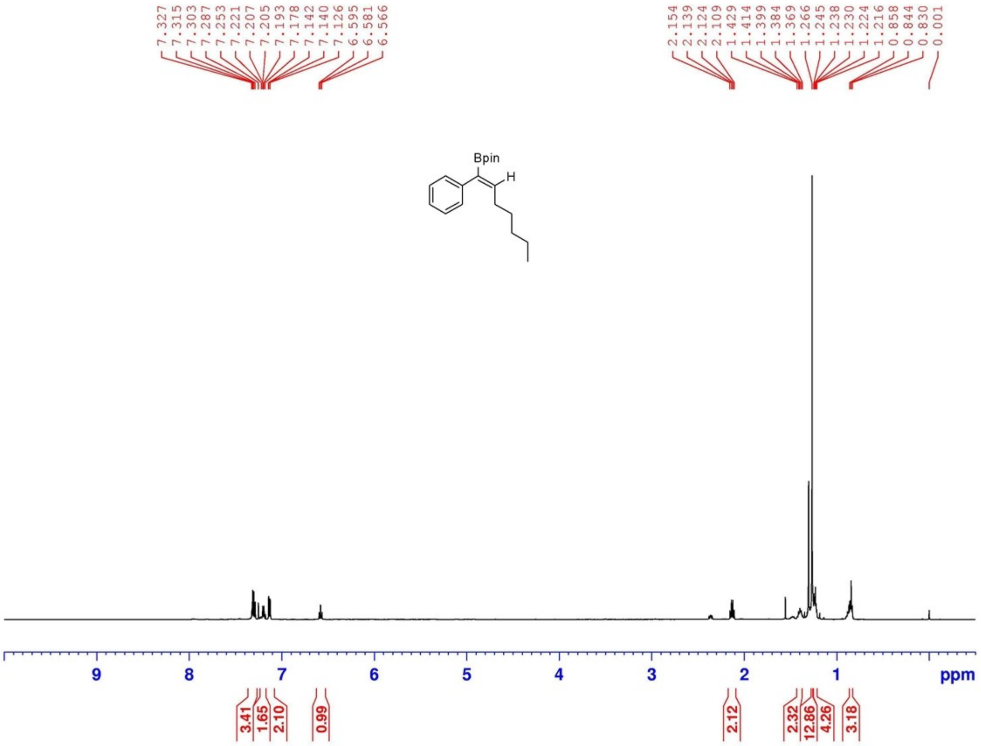

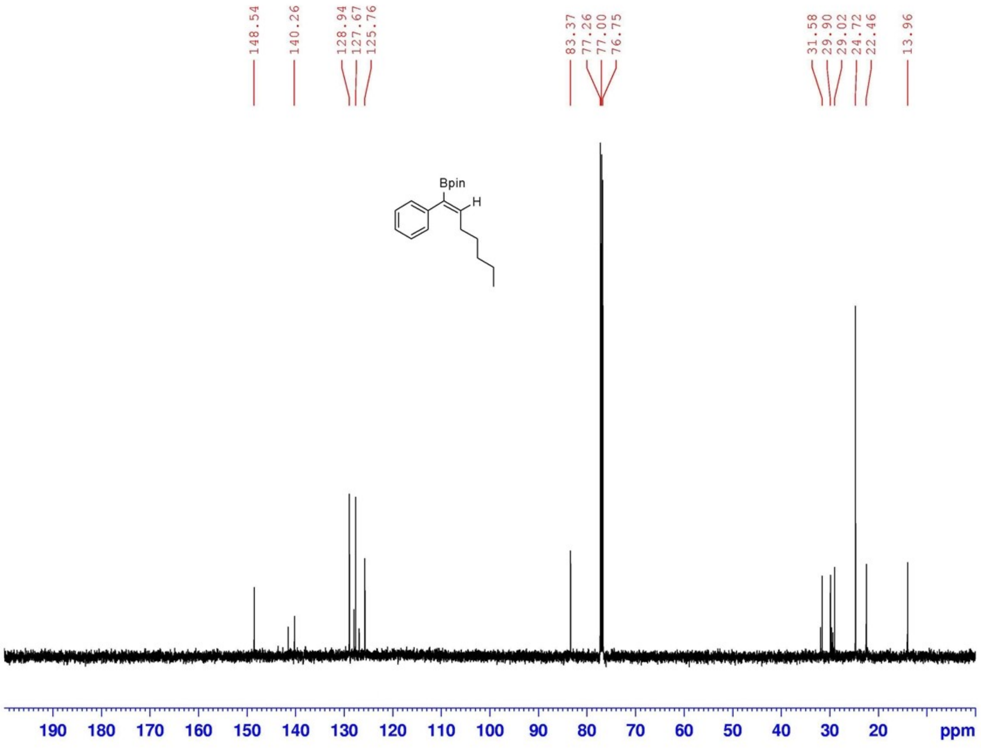

2l ^{1}H and ^{13}C NMR Spectra

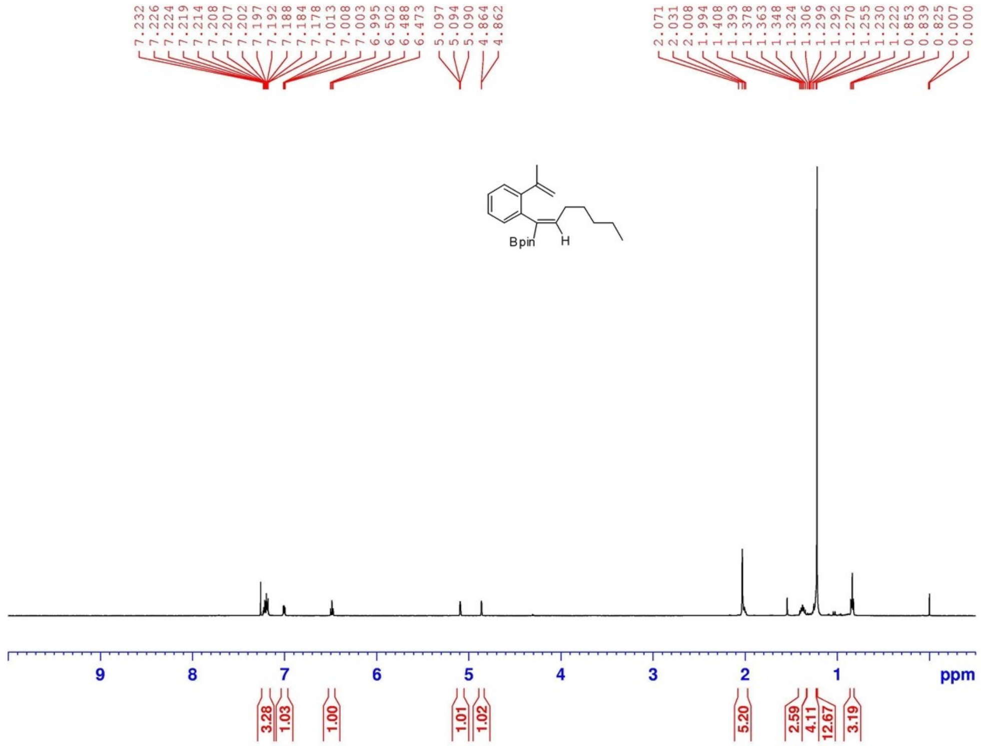

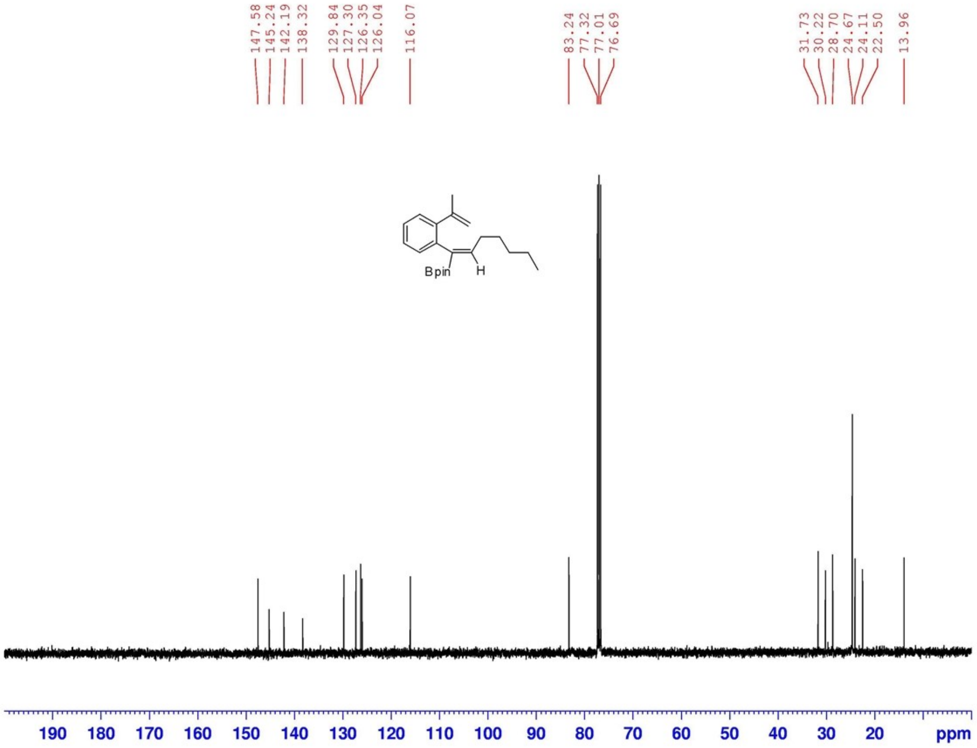

2m ^{1}H and ^{13}C NMR Spectra (The NMR spectra contain isomers)

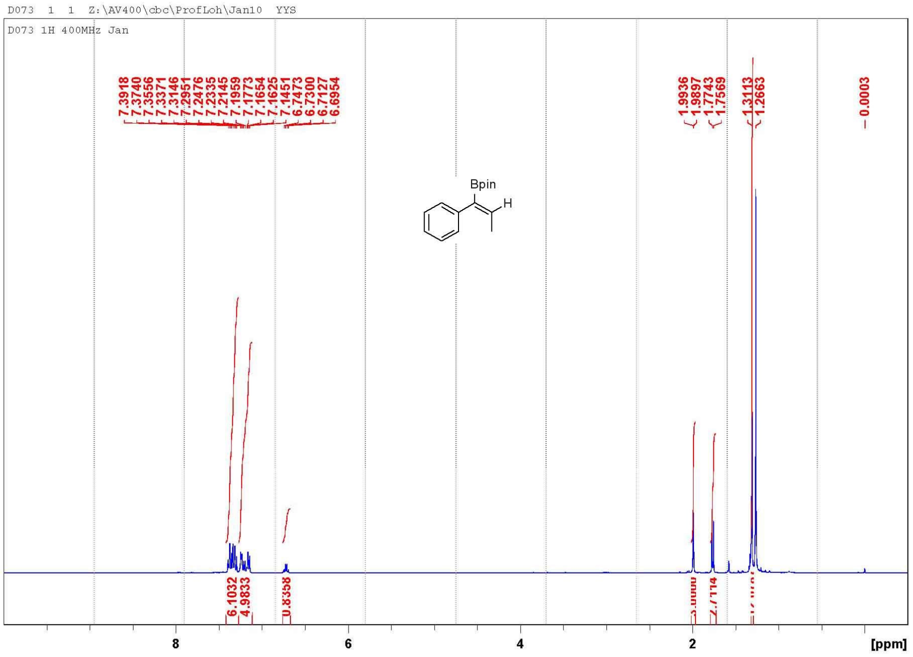

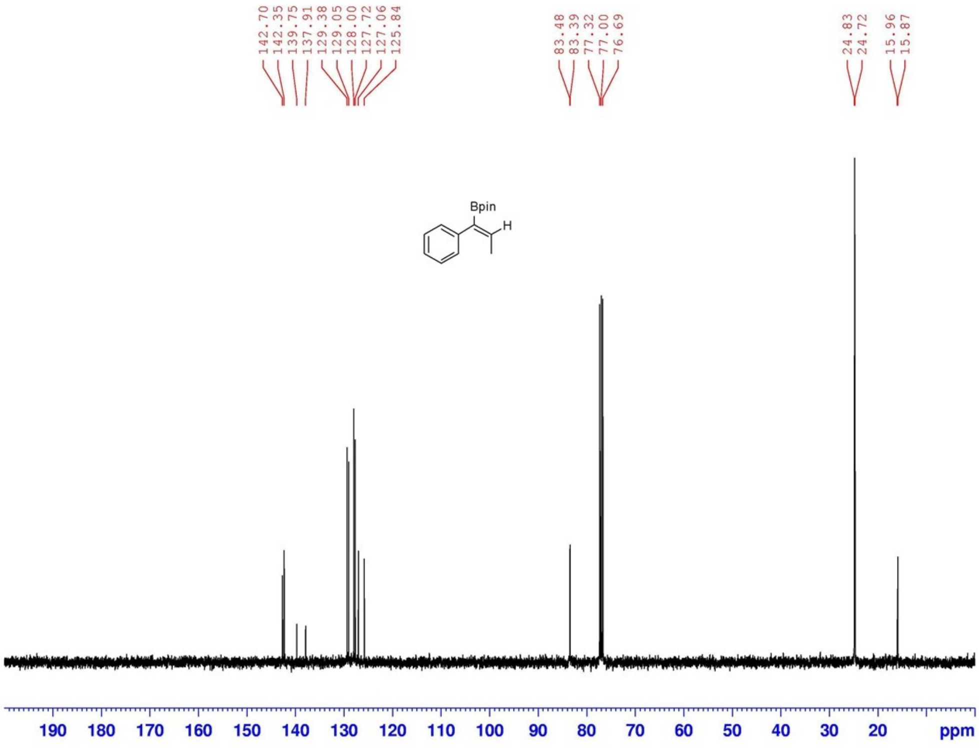

Supplement: RA-012-D1RA09136K-s001 [file RA-012-D1RA09136K-s001.pdf]
